# Design and Synthesis of Novel Heterocyclic-Based 4*H*-benzo[*h*]chromene Moieties: Targeting Antitumor Caspase 3/7 Activities and Cell Cycle Analysis

**DOI:** 10.3390/molecules24061060

**Published:** 2019-03-18

**Authors:** Fawzia F. Alblewi, Rawda M. Okasha, Areej A. Eskandrani, Tarek H. Afifi, Hany M. Mohamed, Ahmed H. Halawa, Ahmed M. Fouda, Al-Anood M. Al-Dies, Ahmed Mora, Ahmed M. El-Agrody

**Affiliations:** 1 Chemistry department, Faculty of Science, Taibah University, Medina 30002, Saudi Arabia; rawdao@yahoo.com (R.M.O.); a.eskandrani@gmail.com (A.A.E.); afifith@yahoo.com (T.H.A.); 2 Chemistry Department, Faculty of Science, Jazan University, Jazan 2097, Saudi Arabia; hanysm83@yahoo.com; 3 Chemistry Department, Faculty of Science, Al-Azhar University, Nasr City, Cairo 11884, Egypt; ahmedhalawa_79@yahoo.com (A.H.H.); autobus78@yahoo.com (A.M.); elagrody_am@yahoo.com (A.M.E.-A.); 4 Chemistry Department, Faculty of Scinece, King Khalid University, P.O. Box 9004, Abha 61413, Saudi Arabia; amfouda@hotmail.com (A.M.F.); nood-mansyaa@hotmail.com (A.-A.M.A.-D.); 5 Biology and Chemistry Department, Al-Qunfudah University College, Umm Al-Qura University, Al-Qunfudah 1109, Saudi Arabia

**Keywords:** fused chromenes and pyrimidines, cell cycle analysis, cell apoptosis, caspase 3/7 activity, DNA fragmentation, cell invasion, and migration, SAR

## Abstract

Novel fused chromenes (**4**,**7**–**11**) and pyrimidines (**12**–**16**) were designed, synthesized, and evaluated for their mammary gland breast cancer (MCF-7), human colon cancer (HCT-116), and liver cancer (HepG-2) activities. The structural identity of the synthesized compounds was established according to their spectroscopic analysis, such as FT-IR, NMR, and mass spectroscopy. The preliminary results of the bioassay disclosed that some of the target compounds were proven to have a significant antiproliferative effect against the three cell lines, as compared to Doxorubicin, Vinblastine, and Colchicine, used as reference drugs. Particularly, compounds **7** and **14** exerted promising anticancer activity towards all cell lines and were chosen for further studies, such as cell cycle analysis, cell apoptosis, caspase 3/7 activity, DNA fragmentation, cell invasion, and migration. We found that these potent cytotoxic compounds induced cell cycle arrest at the S and G2/M phases, prompting apoptosis. Furthermore, these compounds significantly inhibit the invasion and migration of the different tested cancer cells. The structure-activity relationship (SAR) survey highlights that the antitumor activity of the desired compounds was affected by the hydrophobic or hydrophilic nature of the substituent at different positions.

## 1. Introduction

**Figure 1:**
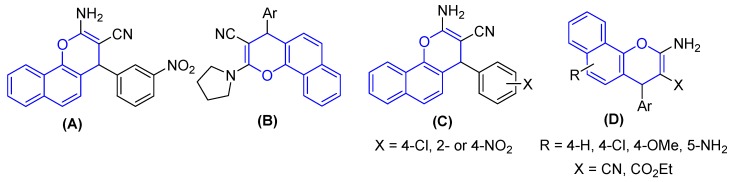
Structures of fused chromene derivatives (blue-highlighted) with biological effects.

Fused chromenes and benzochromenes are notoriously established molecules that exhibit miscellaneous biological activities, such as antibacterial and antifungal activity [1,2,3], being an anticancer agent [4], blood platelet antiaggregating [5], hypolipidemic [6], antioxidant [7,8], antileishmanial [9], vascular-disrupting [10], anticancer, spectroscopic properties, and fluorescence [11,12] activities and effects. Moreover, the fused chromene nuclei have arisen as an encouraging and desirable scaffold for the development of powerful antitumor agents. For instance, *β*-enaminonitrile (**A**) (LY290181) is an effective antiproliferative vehicle for different cell lines, causing a hindrance to the mitosis phase and microtubules [13,14]. The 2-*N*-succinimido derivatives (**B**) display an anti-rheumatic effect [15], while *β*-enaminonitriles (**C**) [16] have effective cytotoxic and apoptotic behaviors against different cell lines: MCF-7, MDA-MB-231, HepG-2, T-47D, SK-N-MC, KB, and PC3. Furthermore, *β*-enaminonitriles/esters (D) [17,18,19,20,21,22] are reported to be one of the most antiproliferative agents, shown in Figure 1.

**Figure 2:**
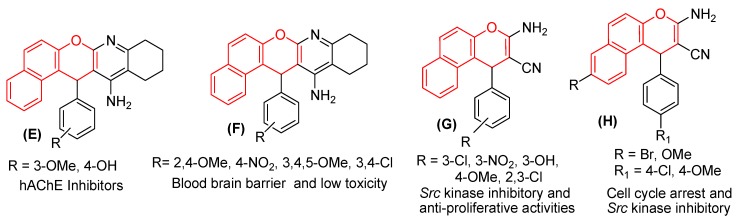
Structures of fused chromenes (red-highlighted) with cytotoxic and apoptotic effects.

Moreover, *β*-enaminonitriles (**E**) (R = 3-OMe, 4-OH) demonstrated high potency for acetylcholinesterase (hAChE) inhibitors in the low micromolar range. The *β*-enaminonitriles compound (**F**) (R = 2,4-OMe, 4-NO2, 3,4,5-OMe, 3,4-Cl) [23] has strong blood–brain barrier permeability profiles, determined in the parallel artificial membrane permeability assay (PAMPA), and experience low toxicity in the HepG-2 cells. The *β*-enaminonitrile compounds (**G**) exhibit antiproliferative and *c-Src* kinase inhibitory activities [24], and the *β*-enaminonitriles (**H**) [25] demonstrate cell cycle arrest at the S and G2/M phases, inducing apoptosis and *c-Src* kinase inhibitory, presented in Figure 2.

**Figure 3:**
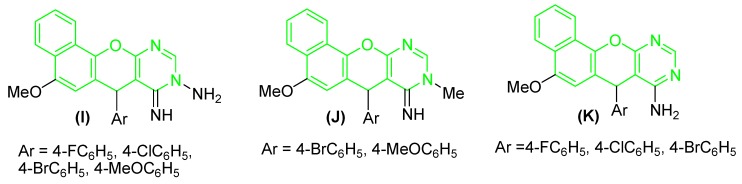
Structure of some fused pyrimidine derivatives (green-highlighted) with cytotoxic and apoptotic effects.

Additionally, fused pyrimidines displayed anticancer characteristics. For instance, the amino–imino compounds (**I**) [20,21,22,26], methylimino compounds (**J**) [20,22], and the amino–imino compounds (**K**) [20,21,22] have higher significant potent antitumor activities against MCF-7, HCT-116, and HepG-2, in comparison to the different standard drugs: Doxorubicin, Vinblastine, and Colchicine, shown in Figure 3.

These findings encouraged us to develop a novel series of chromene and pyrimidine molecules with the aim of discovering their antitumor features [17,18,19,20,21,22,26]. The SAR of the desired molecules stressed the influence of the substituents at different positions on the antitumor activity. In addition, the most potent compounds **7** and **14** were selected to investigate the mechanism of their actions, using various examinations, such as cell cycle analyses, Annexin V assay, caspase 3/7 activity, and DNA fragmentation. Our results revealed that the prepared compounds trigger cancer cell arrest in the S and G2/M phases, and induce caspase dependent apoptosis. The prepared compounds were also able to inhibit cancer cells’ invasion and migration.

## 2. Results and Discussion

### 2.1. Chemistry

**Scheme 1:**
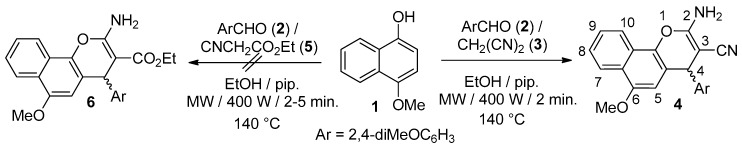
Synthesis of 4*H*-benzo[*h*]chromene derivative **4**.

**Scheme 2:**
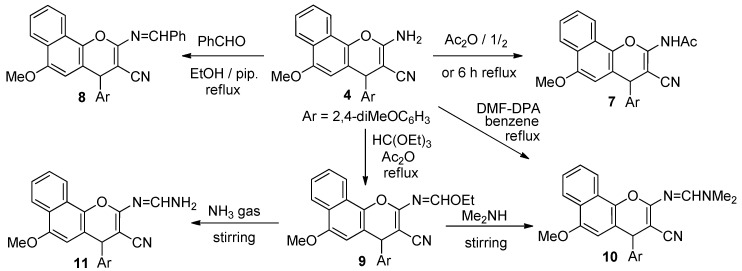
Synthesis of 2-substituted chromenes **7**–**11**.

**Scheme 3:**
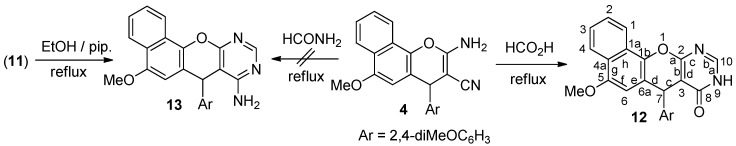
Synthesis of the chromenopyrimidine derivatives, **12** and **13**.

**Scheme 4:**
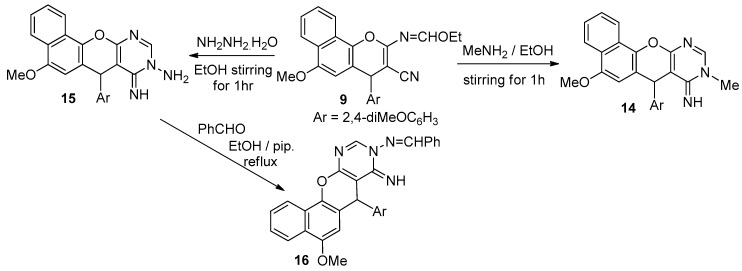
Synthesis of compounds **14**–**16**.

*β*-enaminonitrile is a versatile precursor to the manufacturing of several functionalized heterocycles with a remarkable biological performance [13,14,15,16,17,18,19,20,21,22]. Thus, the synthesis of chromene- and pyrimidine-containing compounds has drawn considerable notice, seen in Scheme 1, Scheme 2, Scheme 3 and Scheme 4. The synthetic methodology was initiated by reacting the 4-methoxy-1-naphthol (**1**), 2,4-dimethoxybenzaldehyde (**2**), and CH_2_(CN)_2_ (**3**), or ethyl cyanoacetate (**5**), in an ethanolic piperidine solution. The reaction was employed for microwave irradiation for 2 min at 140 °C to produce the *β*-enaminonitrile (**4**), however, the formation of the *β*-enaminoester (**6**) was unsuccessful. This result could be attributed to the steric hindrance of the methoxy group at the 2-position of the 2,4-dimethoxybenzaldehyde. Compound **4** displayed optical activity of zero rotation (i.e., it was optically inactive) and, thus, was in the form of a racemic (±) mixture [17,26], as shown in Scheme 1.

The synthesis of the fused chromene derivatives (**7**–**10**) was achieved via the reaction of the *β*-enaminonitrile **4** with different nuclophilic reagents, namely: acetic anhydride, benzaldehyde, triethyl orthoformate, and dimethylformamide-dineopentylacetal (DMF-DPA), while the treatment of **9** with the NH_3_ gas bubbled in methanol at ambient temperature for 1 h, giving the open chain product, 2-aminomethyleneamino derivative **11**, presented in Scheme 2.

The compounds **4** and **9** are beneficial precursors for the construction of an assortment of novel heterocycle-based chromenopyrimidine moieties. Therefore, the condensation of compound **4** with formic acid under reflux yielded pyrimidine-8-one derivative (**12**), while the reaction of **4** with formamide under reflux failed; thus, 8-aminopyrimidine (**13**) was not formed. Alternatively, compound **13** can be attained by the cyclization of **11** in a refluxed ethanolic piperidine solution [27], shown in Scheme 3.

The interface of the imidate **9** with methanamine or hydrazine hydrate allowed the cycloaddition of the methylimino **14** and the amino–imino **15** products. Furthermore, compound **15** reacted with benzaldehyde in a refluxed ethanolic piperidine and generated a Schiff base open product, 9-benzylideneaminopyrimidine **16**, as seen in Scheme 4. 

Moreover, the methine protons of compounds **4**, **7**–**11**, and **12**–**16** are chiral centers. The identity of all the new compounds were verified, according to their spectral data, IR, ^1^H NMR, ^13^C NMR, and MS data (see Experimental section and Supplementary Materials).

### 2.2. Biological Evaluation

#### 2.2.1. In Vitro Cytotoxic Activity

**Figure 4:**
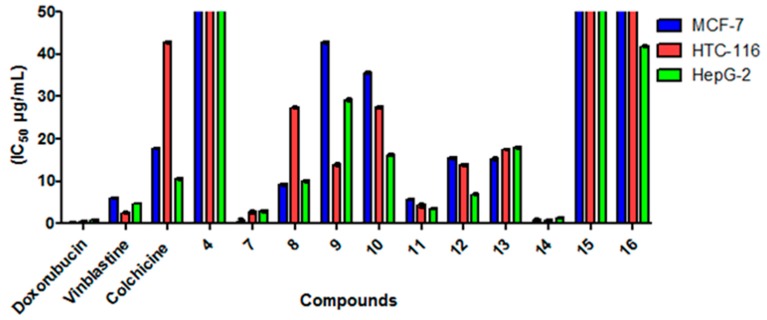
IC_50_ values for MCF-7, HCT-116, and HepG-2 tumor cells after 24 h treatment with fused chromenes **4, 7**–**11** and pyrimidines **12**–**16**.

The in vitro antiproliferative performance of compounds **4**, **7**–**11**, and **12**–**16** towards breast cancer MCF-7, human colon cancer HCT-116, and liver cancer HepG-2 cell lines was explored, as shown in Figure 4 and Table 1. The judicious choice of the cell lines and standard drugs was instigated by the asserted anticancer behavior of a number of benzochromene and chromenopyrimidine molecules [13,14,15,16,17,18,19,20,21,22,23,24,25,26]. Their cytotoxic activities were evaluated via the 2-(4,5-dimethylthiazol-2-yl)-3,5-diphenyl-2*H*-tetrazol-3-ium bromide (MTT) colorimetric assay [28,29], and the obtained IC_50_ values were in comparison to the values of the reference cytotoxic drugs: Doxorubicin, Vinblastine, and Colchicine. 

From the attained results in Table 1, compounds **7**, **14**, and **11** were discovered to be the most potent derivatives of all the tested compounds against the MCF-7 cancer cell line, as it was 8.7, 6.8, and 1.1 times, and 25.3, 19.7, and 3.1 times more active than both the standard drugs Vinblastine and Colchicine, respectively, while compounds **8**, **13**, and **12** exhibited strong activity against the MCF-7 cancer cell, in comparison to Colchicine. Additionally, compound **14** was the most active candidate against HCT-116, as it was 3.3 and 53.5 times more active than both standard drugs, Vinblastine and Colchicine, respectively, and compound **7** was equipotent to Vinblastine. In the meantime, compounds **7**, **11**, **12**, **9**, **13**, **8**, and **10** displayed strong activity against the HCT-116 cancer cell, due to them being 15.3, 10.0, 3.1, 3.1, 2.5, 1.6, and 1.6 times more active than Colchicine. On the other hand, the cytotoxicity evaluation in the HepG-2 cell line divulged that compounds **14**, **7**, and **11** were the most active congener against HepG-2 with 3.3, 1.5, and 1.2 times, and 7.6, 3.5, and 2.7 times more activity, in comparison to Vinblastine and Colchicine, respectively. Additionally, compounds **12** and **8** exhibited strong activity against the HepG-2 cancer cell with them being 1.5 and 1.1 times more active than Colchicine. Compound **14** was almost equipotent to Doxorubicin against the MCF-7, HCT-116, and HepG-2 cancer cell lines.

#### 2.2.2. Cell Cycle Analysis 

**Figure 5:**
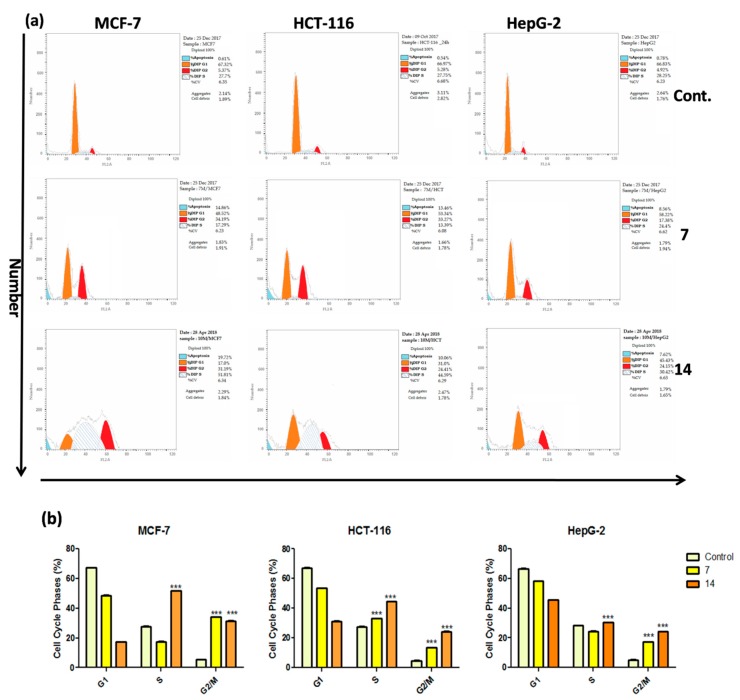
(**a**) Cell cycle profile of MCF-7, HCT-116, and HepG-2 cancer cells were analyzed with PI. The plot shows the distribution of cells in G1 (orange) and G2/M (red) phases of the cell cycle, and cells in the S phase are located between the two peaks, represented by the hatched curve. (**b**) Bar charts, representing the percentage of cell populations in MCF-7, HCT-116, and HepG-2 cells, were treated with compounds **7** and **14** (4 μg/mL) for 24 h. The data are expressed as the mean ± SD of three independent experiments in triplicate. Significances are shown in comparison to control cells (*** *p* < 0.001).

Numerous anticancer molecules employ their impact via blocking cell cycle progression, inducing apoptosis, or the merged effect of both. [30,31]. To verify the causal relation of cell proliferation inhibition and cell cycle arrest, the cell cycle distribution was probed, using the Propidium Iodide Flow Cytometry Kit assay. The cell cycle phase distribution, which was examined, showing the collected propidium iodide fluorescence intensity on the FL2, is displayed in Figure 5a. Compound **7** experienced an increased cell number at the G2/M phases after 24 h, accompanied by a decreased cell number at the S and G1 phases in the MCF-7 and HepG-2 cells. However, in the case of the HCT-116 cells, compound **7** demonstrated cancer cell arrest at both the S and G2/M phases. Furthermore, compound **14** exhibited a significant increased cell number at the S and G2/M phases, with a decreased cell number at the G1 phase (Figure 5b). The G2 checkpoint inhibits cells from entering mitosis, and this is commonly observed in cells that have been exposed to DNA-damaging agents and the topoisomerase blockers [32].

#### 2.2.3. Cell Apoptosis

**Figure 6:**
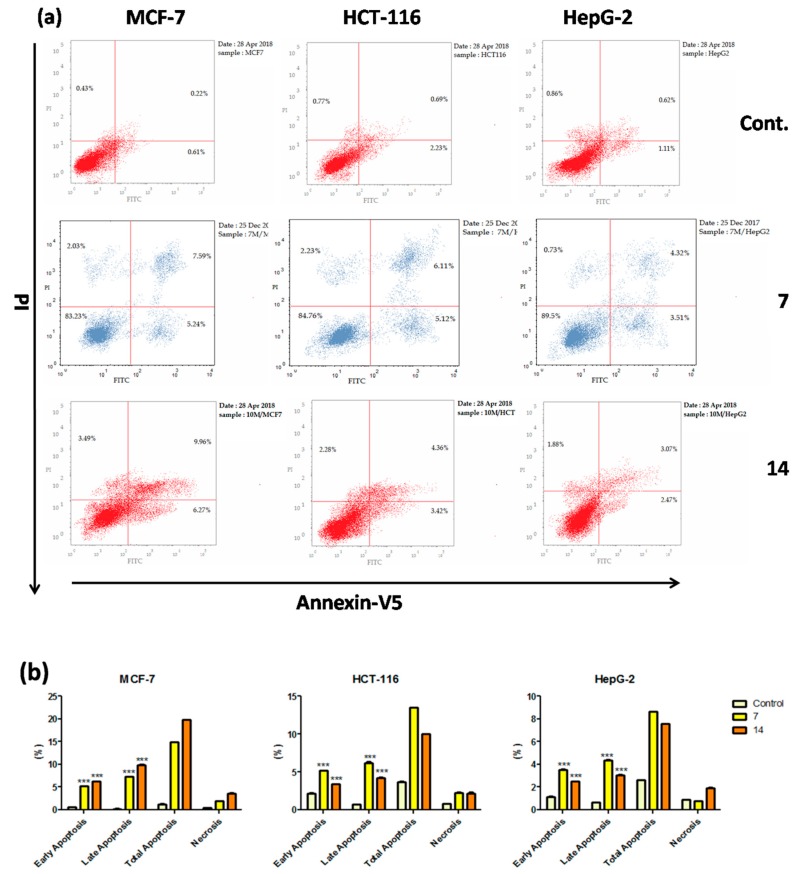
(**a**) Dot plot of Annexin V/PI stained cells treated with the indicated drugs for 24 h. (**b**) Apoptosis percentage of MCF-7, HCT-116, and HepG-2 cells after incubation with compounds **7** and **14** (4 μg/mL) for 24 h. The data are expressed as the mean ± SD of three independent experiments in triplicate. Significances are shown in comparison to control cells (*** *p* < 0.001).

Further investigation on the mechanism, dealing with apoptotic cell death and induced by compounds **7** and **14** on the different treated cells, was explored. This study was performed using the Annexin V assay, which is beneficial for the detection of translocated phosphatidylserine (PS), a hallmark of apoptosis [33]. The dot plot flow cytometry data of the stained cells with the Annexin V-FITC and PI is shown in Figure 6a. After 24 h of exposure, the MCF-7, HCT-116, and HepG-2 cells had undergone early and late apoptosis when treated with compound **7** or **14**, in comparison to the untreated control cells. A considerable increase in the number of apoptotic cells was also observed in all cells after exposure to compound **7** or **14** (Figure 6b). Necrosis was not observed in any of the treatment conditions, suggesting cell death occurred primarily through the induction of apoptosis.

#### 2.2.4. Caspase 3/7 Activity 

**Figure 7:**
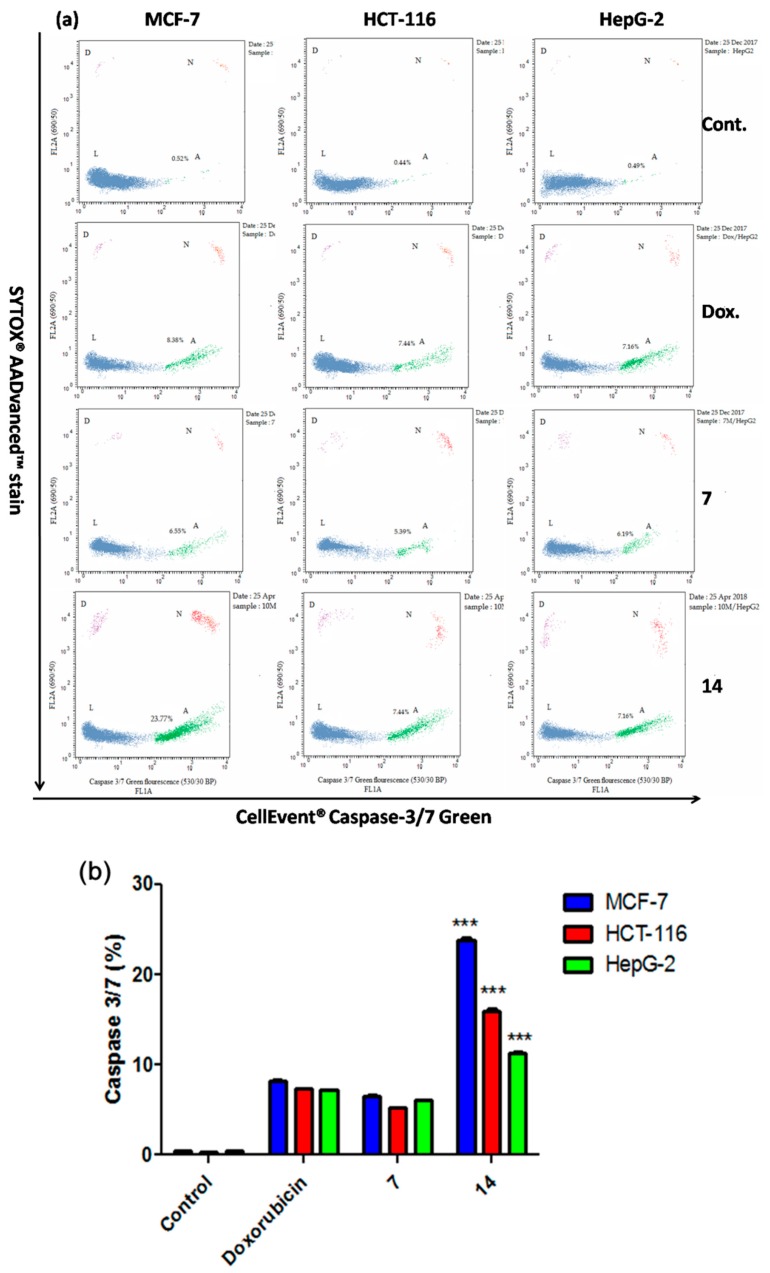
(**a**) Detected caspase activity in cancer cells was treated with the indicated drugs for 24 h, using the CellEvent^®^ Caspase-3/7 Green Flow Cytometry kit. Fluorescence emission was collected, using a 530/30 bandpass filter for the CellEvent^®^ reagent and a 690/50 bandpass filter for the SYTOX^®^ AADvanced™ stain (also provided in the kit). L, viable cells; A, apoptotic cells; N, necrotic cells;. and D, dead cells. (**b**) Caspase-3/7 activity percentage of MCF-7, HCT-116, and HepG-2 cells after incubation with compounds **7** and **14** (4 μg/mL) for 24 h. The data are expressed as the mean ± SD of three independent experiments in triplicate. Significances are shown in comparison to doxorubicin-treated cells (*** *p* < 0.001).

Apoptosis is a type of programmed cell death that is managed by the members of the caspase family of cysteine proteases. Procaspases are activated in response to the diverse cell death stimuli, ultimately producing an amplifying, irreversible proteolytic cascade [34]. To further establish the apoptotic procedure generated by the tested compounds, the effector caspases’ activity was assessed, using the Cell Event™ Caspase-3/7 Green Detection kit (Figure 7a). All treated cells have a higher percentage of apoptotic cells than the basal level of apoptosis seen in the control cells. The levels of caspase-3/7 of all the tested cells, treated with compounds 7, were more or less equivalent to the reference drug Doxorubicin. Moreover, compound **14** significantly amplified the level of caspase-3/7 in all the tested cells, which were double the levels achieved by Doxorubicin (Figure 7b). Taken together, these data revealed that compound **7** and **14** triggered cell apoptosis by activating the effector caspases 3 and 7.

#### 2.2.5. DNA Fragmentation

**Figure 8:**
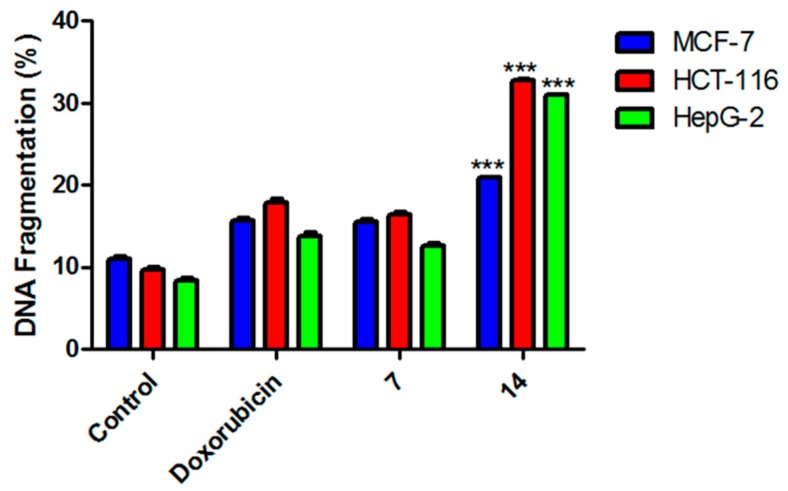
DNA fragmentation percentage of MCF-7, HCT-116, and HepG-2 cells after incubation with compounds **7** and **14** (4 μg/mL) for 24 h. The data are expressed as the mean ± SD of three independent experiments in triplicate. Significances are shown in comparison to doxorubicin treated cells (*** *p* < 0.001).

Caspases play principal roles in the induction of DNA fragmentation, which is the most common characteristic biochemical event in apoptosis pathways [35]. The DNA fragmentation was quantitatively determined, using the diphenylamine (DPA) reagent. The results were attained from the relative quantity of DNA fragments, integrated with the caspases’ activity results acquired from before. We found that compound **7** showed more or less the same DNA fragmentation percentage as Doxorubicin in all the tested cells. These results also demonstrated the significant influence of compound **14** on DNA fragmentation, which was almost double the results obtained by Doxorubicin in all the tested cells (Figure 8). Overall, compounds **7** and **14** encourage apoptosis through DNA fragmentation and executioner caspase-3/7 activation.

#### 2.2.6. Cell Invasion and Migration

**Figure 9:**
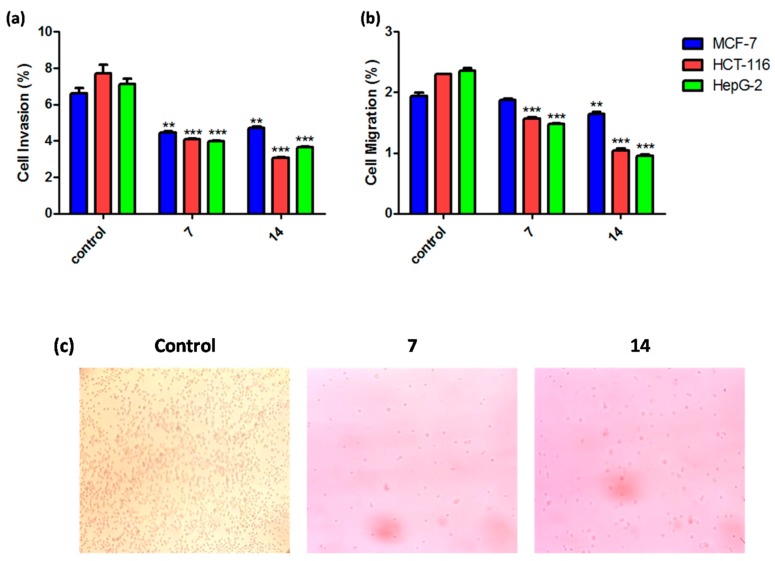
Cell (**a**) invasion and (**b**) migration percentage of MCF-7, HCT-116, and HepG-2 cells after incubation with compounds **7** and **14** (4 μg/mL) for 24 h. The data are expressed as the mean ± SD of three independent experiments in triplicate. Significances are shown in comparison to control cells (** *p* < 0.01; *** *p* < 0.001). (**c**) Matrigel invasion assay in MCF7 control cells, co-cultured cells with compounds **7** and **14**. Cells that crossed the Matrigel-coated filter were fixed, stained, and counted. Six random microscopic fields were counted for each group.

In this study, the cell cycle arrest at the G2/M phases raised the possibility that the tested compounds might manipulate cell invasion and migration. Compounds **7** and **14** exhibited a substantial decrease in the cell invasion and cell migration percentage in all the tested cells, compared to the untreated control cells (Figure 9). Microtubules play an imperative role in cell migration and invasion through numerous pathways [36]. The possible effect of the tested compounds on the microtubules might be the reasons for the demonstrated data.

All the results reveal that the potent antitumor effect of compound **7** is due to the inhibition of the G2/M cell cycle checkpoint. This action is associated with the induction of the executioner caspase 3/7 that induces DNA fragmentation, ending with apoptosis, as shown with the externalization of phosphatidylserine (PS), which is a hallmark of apoptosis. The data has also highlighted the possible effect of compound **7** on DNA damage, topoisomerase enzyme inhibition, and the disruption of the microtubules assembly, which may be the first sight of action [37,38]. Finally, compound **7** may be useful as a novel potent anticancer drug in the future.

#### 2.2.7. SAR Studies

The preliminary SAR study fixated on the impact of replacement at the 2-position or the fused rings at the 2-, 3-positions on the antitumor activities of the synthesized compounds. In an assessment of the cytotoxic activities of the two sets of fused chromenes (**4**, **7**–**11**) and pyrimidines (**12**–**16**) against the MCF-7 cell line, we found that the highest growth inhibitory effect of the first series **4**, **7**–**11** was associated with the presence of the hydrophobic groups, –NHAc and -N=CHNH_2_, at the 2-positon of the 4H-benzo[h]chromene moiety for compounds **7** and **11** with IC_50_ values of 0.7 and 5.8 µg/mL, which is considered excellent activity, relative to Vinblastine and Colchicine (IC_50_ = 6.1 and 17.7 μg/mL), respectively. Meanwhile, concerning the inhibitory effect of the second series **12**–**16**, the highest growth inhibitory effect was associated with the existence of a fused pyrimidine ring with hydrophobic groups (=NH-8, -Me-9) at the 2-, 3-positions of the chromene moiety for compound **14** with an IC_50_ value of 0.9 µg/mL, in comparison to Vinblastine (IC_50_ = 6.1 μg/mL) and Colchicine (IC_50_ = 17.7 μg/mL), respectively. Thus, the strong lipophilic characteristic of the molecule performs a crucial role in generating the antitumor effect. From this perspective, the existence of the hydrophobic substituent in the pyrimidine moiety would be much more crucial for such activity than in the 4H-benzo[h]chromene moiety.

Furthermore, compound **7** and **11** (IC_50_ = 2.8 and 4.3 μg/mL), bearing hydrophobic substituents –NHAc and -N=CHNH_2_ at the 2-position for the first series, exhibited an increase in activity against the HCT-116 cancer cell, compared to Vinblastine, Colchicine, and other derivatives. The introduction of a pyrimidine ring at the 2-, 3-positions of compound **4** with hydrophobic groups (=NH-8, -NH_2_-9) for compound **14** (IC_50_ = 0.8 μg/mL) resulted in a remarkable enhancement of the potency against the HCT-116 cancer cell, in evaluation against Vinblastine and Colchicine (IC_50_ = 2.6 and 42.8 μg/mL). On the other hand, compounds **7**, **8**, **9**, and **10**, and **12** and **13**, for the first and second series, respectively, exhibited efficient cytotoxicity in comparison to Colchicine, suggesting that the pyrimidine nucleus at the 2-, 3-positions with the hydrophobic group at the 8-, 9-positions was imperative for the activities towards the HCT-116 cancer cell, rather than the chromene nucleus with the hydrophobic group at the 2-position.

Concerning the activity against HepG-2, compounds **7** and **11** for the first series and compound **14** for the second series were the most active analogs through this study with IC_50_ values of 3.0, 3.7, and 1.4 μg/mL, respectively, in comparison to the reference drugs, Vinblastine and Colchicine (IC_50_ = 4.6 and 10.6µg/mL). These results imply that the introduction of the hydrophobic groups (=NH-8, -Me-9) in the pyrimidine moiety was indispensable for the activity against HepG-2, compared to the hydrophobic groups –NHAc and -N=CHNH_2_ at the 2-positon of the chromene moiety.

In addition, compound **14** had the strongest impact against MCF-7, HCT-116, and HepG-2 as evaluated against Doxorubicin. Finally, we can deduce that the substitution pattern at the 2-position or fused rings at the 2, 3-positions on the synthesized chromene and pyrimidine moieties is a crucial element for antitumor activity. The incorporation of the pyrimidine rings at the 2-, 3-positions with the hydrophobic groups as (=NH-8, -Me-9) or (-N=CHNH_2_) at the 2-position of the chromene nucleus is favorable and greatly enriches the activity, more so than the other hydrophobic and hydrophilic groups.

## 3. Materials and Methods

### 3.1. General Information

Commercial-grade solvents and reagents were purchased from Sigma–Aldrich (St. Louis, MO, USA) and used without further purification. Melting points were measured with a Stuart Scientific (UK) apparatus and are uncorrected. IR spectra were determined as KBr pellets on a Jasco FT/IR 460 plus spectrophotometer (Jasco, Japan). The optical activities of the synthesized compounds were measured using a CARL ZEISS JENA 267628 polarimeter. ^1^H-NMR, ^13^C-NMR, ^13^C-NMR/APT, and ^13^C-NMR/DEPT spectra were recorded using a Bruker AV 500 MHz spectrometer (Bruker, Billerica, MA, USA). Chemical shifts (δ) are expressed in parts per million (ppm). The microwave synthesis was performed using a mono-mode Milestone Sr1 device (Milestone, Shelton, CT, USA), while the MS spectra were measured using a Shimadzu GC/MS-QP5050A spectrometer (Shimadzu, Japan). Elemental analyses were carried out at the Regional Centre for Mycology & Biotechnology (RCMP), Al-Azhar University, Cairo, Egypt and the results were within ±0.25%. Analytical thin layer chromatography (TLC) on silica gel precoated F_254_ (Merck, Billerica, MA, USA) plates was used to check the purity of the compounds.

### 3.2. Synthesis

*2-Amino-4-(2,4-dimethoxyphenyl)-6-methoxy-4H-benzo[h]chromene-3-carbonitrile* (**4**)

Chromene compound **4** was prepared according to the previously reported procedure [39]. 

*2-Acetylamino-4-(2,4-dimethoxyphenyl)-6-methoxy-4H-benzo[h]chromene-3-carbonitrile* (**7**). *β*-enaminonitrile **4** (3.88 g, 0.01 mol) was refluxed in acetic anhydride (20 mL) for 0.5 or 6 h. Upon the removal of the solvent, the resulting solid was collected, washed with cooled methanol, filtered, dried, and recrystallized from ethanol to provide **7** as a colorless solid, yield: 97%, m.p. 167–168 °C; C_25_H_22_N_2_O_5_ (430.45); calcd; % C: 69.76, % H: 5.15, % N: 6.51; found; % C: 69.81, % H: 5.18, % N: 6.57.

*2-Benzylideneamino-4-(2,4-dimethoxyphenyl)-6-methoxy-4H-benzo[h]chromene-3-carbonitrile* (**8**). A 2 h. refluxed reaction of *β*-enaminonitrile 4 (3.88 g, 0.01 mol), benzaldehyde (1.06 g, 0.01 mol) and piperidine (0.5 mL) in 20 mL ethanol produced compound **8**, which was recrystallized from ethanol/benzene as a yellow solid, yield: 63%, m.p. 215–216 °C; C_30_H_24_N_2_O_4_ (476.52); calcd; % C: 75.61, % H: 5.08, % N: 5.88; found; % C: 75.55, % H: 4.99, % N: 5.79.

*2-Ethoxymethyleneamino-4-(2,4-dimethoxyphenyl)-6-methoxy-4H-benzo[h]chromene-3-carbonitrile* (**9**). A combination of *β*-enaminonitrile 4 (3.88 g, 0.01 mol) and triethyl orthoformate (1.48 g, 0.01 mol) in a boiled Ac_2_O (30 mL) for 2 h, followed by a recrystallization from benzene to afford compound **9** as a yellow solid, yield: 91%, m.p. 186–187 °C; C_26_H_24_N_2_O_5_ (444.48); calcd; % C: 70.26, % H: 5.44, % N: 6.30; found; % C: 70.30, % H: 5.48, % N: 6.41.

*2-Dimethylaminomethyleneamino-4-(2,4-dimethoxyphenyl)-6-methoxy-4H-benzo[h]chromene-3-carbonitrile* (**10**) Method (a): *β*-enaminonitrile 4 (3.88 g, 0.01 mol) with dimethylformaide-dineopentylacetal (DMF-DPA) (2.31 g, 0.01 mol) and benzene (30 mL) was refluxed for 3 h. The isolated solid was recrystallized from benzene to give **10** as a colorless solid, yield: 90%, m.p. 206–207 °C; C_26_H_25_N_3_O_4_ (443.49); calcd; % C: 70.41, % H: 5.68, % N: 9.47; found; % C: 70.48, % H: 5.71, % N: 9.50. Method (b): The imidate 9 (4.44 g, 0.01 mol) and dimethylamine (0.45 g. 0.01 mol) in methanol (30 mL) was stirred at room temperature for 1 h then left overnight to provide **10** (m.p., mixed m.p., identical IR and MS spectrum).

*2-Aminomethyleneamino-4-(2,4-dimethoxyphenyl)-6-methoxy-4H-benzo[h]chromene-3-carbonitrile* (**11**). Compound **11** was prepared from the imidate **9** (4.44 g, 0.01 mol) and NH_3_ gas bubbled in methanol (30 mL), according to the procedure described for compound **10** (Method b). Compound **11** was recrystallized from ethanol as a colorless solid, yield: 85%, m.p. 230–231 °C; C_24_H_21_N_3_O_4_ (415.44); calcd; % C: 69.39, % H: 5.10, % N: 10.11; found; % C: 69.29, % H: 5.00, % N: 10.00.

*7-(2,4-Dimethoxyphenyl)-5-methoxy-7H,9H-benzo[h]chromeno[2,3-d]pyrimidin-8-one* (**12**). A 3–5 h refluxing of *β*-enaminonitrile **4** (3.88 g, 0.01 mol) and formic acid (4.6 g, 1 mol), followed by the removal of excess formic acid, resulted in the formation of **12**, which was recrystallized from ethanol/benzene as a colorless solid, yield: 83%, m.p. 209–210 °C; C_24_H_20_N_2_O_5_ (416.43); calcd; % C: 69.22, % H: 4.84, % N: 4.84; found; % C: 69.33, % H: 4.92, % N: 6.82.

*8-Amino-7-(2,4-dimethoxyphenyl)-5-methoxy-7H-benzo[h]chromeno[2,3-d]pyrimidine* (**13**). Boiling compound **11** (4.15 g, 0.01 mol) in a pipridine/ethanol solution for 2 h provided compound **13**, which was recrystallized from ethanol as a colorless solid, yield: 74%, m.p. 220–221 °C; C_24_H_21_N_3_O_4_ (415.44); calcd; % C: 69.39, % H: 5.10, % N: 10.11; found; % C: 69.30, % H: 5.00, % N: 10.02.

*7-(2,4-Dimethoxyphenyl)-5-methoxy-8-imino-9-methyl-7H-benzo[h]chromeno[2,3-d]pyrimidine* (**14**). Compound **14** was synthesized via the reaction of the imidate **9** (4.44 g, 0.01 mol) and methanamine (0.31g, 0.01 mol) in methanol (30 mL), pursuing a similar approach to the one for compound **11**. Compound **14** was recrystallized from ethanol/benzene as a colorless solid, yield: 65%, m.p. 223–224 °C; its MS (*m*/*z*), 429 (M^+^, 50.8) with a base peak at 292 (100); C_25_H_23_N_3_O_4_ (429.47); calcd; % C: 69.92, % H: 5.40, % N: 9.78; found; % C: 70.00, % H: 5.50, % N: 9.82.

*9-Amino-7-(2,4-dimethoxyphenyl)-5-methoxy-8-imino-7H-benzo[h]chromeno[2,3-d]pyrimidine* (**15**). The replacing of methanamine in the aforementioned procedure with hydrazine hydrate (0.5 g, 0.01 mol) supplied compound **15**, which was recrystallized from benzene as a colorless solid, yield: 73%, m.p. 198–199 °C; C_24_H_22_N_4_O_4_ (430.46); calcd; % C: 66.97, % H: 5.15, % N: 13.02; found; % C: 70.00, % H: 5.24, % N: 12.94.

*9-Benzylideneamino-7-(2,4-dimethoxyphenyl)-5-methoxy-8-imino-7H-benzo[h]chromeno[2,3-d]pyrimidine* (**16**). A 2 h. refluxed combination of 15 (4.30 g, 0.01 mol) and benzaldehyde (1.06 g, 0.01 mol) in ethanol (30 mL) and piperidine (0.5 mL) produced the open chain product **16** after a recrystallization from ethanol/benzene as a yellow solid, yield: 94%, m.p. 220–221 °C; C_31_H_26_N_4_O_4_ (518.56); calcd; % C: 71.80, % H: 5.05, % N: 10.80; found; % C: 71.77, % H: 5.01, % N: 10.76.

### 3.3. Biological Screening

#### 3.3.1. Cell Culture

The tumor cell lines, breast adenocarcinoma (MCF-7), human colon carcinoma (HCT-116), and hepatocellular carcinoma (HepG-2), were obtained from the American Type Culture Collection (ATCC, Rockville, MD). The cells were grown on an RPMI-1640 medium, supplemented with 10% inactivated fetal calf serum and 50 µg/mL gentamycin. The cells were maintained at 37 °C in a humidified atmosphere with 5% CO_2_ and were subcultured two to three times a week.

#### 3.3.2. Cytotoxicity Evaluation using Viability Assay

The cytotoxic activity was appraised, using the 2-(4,5-dimethylthiazol-2-yl)-3,5-diphenyl-2*H*-tetrazol-3-ium bromide (MTT) colorimetric assay, as reported previously [28,29].

#### 3.3.3. Cell-Cycle Analysis

The effect of compounds **7** and **14** on the DNA content by cell cycle progression were evaluated, utilizing MCF-7, HCT, and HepG-2 cells, as reported previously [40].

#### 3.3.4. Apoptosis Analysis by Flow Cytometry

The apoptosis assay of compounds **7** and **14** was performed with an Annexin V-FITC/PI double-staining apoptosis detection kit (K101, Biovison), as reported previously [33].

#### 3.3.5. Caspase 3/7 Activity Assay

The caspase activity was measured after cell incubation for 24 h with compounds **7** and **14** (4 μg/mL), using a CellEvent™ Caspase-3/7 Green Flow Cytometry Assay Kit (C10427, Invitrogen), as reported previously [34].

#### 3.3.6. DNA Fragmentation

The DNA fragmentation of compounds **7** and **14** was quantitatively determined, utilizing the diphenylamine (DPA) reagent, according to the method of Boraschi and Maurizi (1998) [41]. The optical density was determined at 600 nm in the S (supernatant before cell lysis), T (pellet with the intact DNA), and B (supernatant with fragmented DNA) fractions, respectively. The percentage of the DNA fragmentation was calculated as follows:

% Fragmented DNA = S + T/S + T + B × 100

#### 3.3.7. Cell Invasion Assay

Deregulated cell migration with the set compounds was performed, exploiting the Transwell chamber with a non-coated membrane (96-well HTS Transwell Permeable Supports with 8 μm pores, Cat. No. 3374, Corning, Life Sciences), as reported previously [36].

#### 3.3.8. Statistics

The statistical analysis was performed by the GraphPad Prism 5.01 (GraphPad software, Inc., San Diego, CA, USA). The experiments were analyzed, using one- or two-way ANOVA, followed by a Tukey post hoc test. The statistical significance indicated that * *p* ≤ 0.05, ** *p* ≤ 0.01, and *** *p* ≤ 0.001.

## 4. Conclusions

Several fused chromenes (**7**–**11**) and pyrimidines (**12**–**16**) were synthesized, via the exploitation of *β*-Enaminonitrile **4** as a precursor. The pharmacological analysis was undertaken to assess the effect of the substituents and the pyrimidine rings on the antitumor activities. Most of the synthesized compounds exhibited effective antitumor activities against the three tumor cell lines: (MCF-7), (HCT-116), and (HepG-2). Furthermore, the presence of the pyrimidine rings at the 2, 3-positions with the hydrophobic group as (=NH-8, -Me-9) or (-N=CHNH_2_) at the 2-position of the chromene nucleus significantly enhances activity, in comparison to the other hydrophobic and/or hydrophilic groups. In summary, compounds **7** and **14** exhibit potent cytotoxic and antiproliferative effects on the three cancer cells through the caspase-dependent apoptosis mechanism. DNA damage, the inhibition of the Topoisomerase enzyme, or the disruption of the microtubules’ assembly may be the reason for the induced S & G2/M-phases’ arrest in this study, which will require further studies in the future.

## Figures and Tables

**Table 1:**
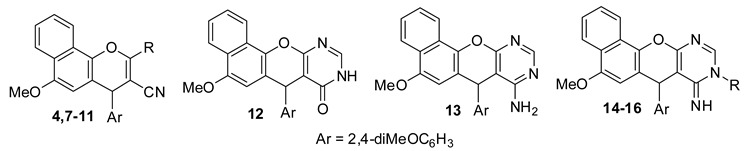
Cytotoxic activities of target compounds against MCF-7, HCT-116, and HepG-2 tumor cells.

Compound	R	IC_50_ (µg/mL) ^a^		
		MCF-7	HCT-116	HepG-2
**4**	NH_2_	w	w	w
**7**	NHAc	0.7 ± 0.23	2.8 ± 0.23	3.0 ± 0.11
**8**	N=CHPh	9.3 ± 0.14	27.4 ± 0.13	10.1 ± 0.15
**9**	N=CHOEt	42.9 ± 0.11	14.0 ± 0.3	29.2 ± 0.3
**10**	N=CHNMe_2_	35.6 ± 0.14	27.5 ± 0.11	16.2 ± 0.1
**11**	N=CHNH_2_	5.8 ± 0.02	4.3 ± 0.32	3.7 ± 0.03
**12**	-	15.6 ± 0.13	13.9 ± 0.17	7.0 ± 0.14
**13**	-	15.3 ± 0.21	17.5 ± 0.04	18.0 ± 0.05
**14**	Me	0.9 ± 0.21	0.8 ± 0.12	1.4 ± 0.1
**15**	NH_2_	w	w	w
**16**	N=CHPh	w	w	41.9
Vinblastine	-	6.1 ± 0.03	2.6 ± 0.08	4.6 ± 0.01
Colchicine	-	17.7 ± 0.01	42.8 ± 0.02	10.6 ± 0.04
Doxorubicin	-	0.4 ± 0.01	0.5 ± 0.02	0.9 ± 0.04

^a^ IC_50_ values expressed in µg/mL as the mean values of triplicate wells from at least three experiments are reported as the mean ± standard error. w = weak activity (IC_50_ ≥ 75 g/mL).

## Supplementary Materials

The following are available online. Spectral data of 2-substituted 4*H*-benzo[*h*]chromene derivatives (4–11, 13, 14); Spectral data of 7*H*-benzo[*h*]chromeno[2,3-*d*]pyrimidine derivatives (12–16).
