# Supplementary material for: Design and Synthesis of Novel Heterocyclic-Based 4H-benzo[h]chromene Moieties: Targeting Antitumor Caspase 3/7 Activities and Cell Cycle Analysis
Source: Molecules. 2019 Mar 18;24(6):1060. doi: 10.3390/molecules24061060 (PMC6471608; doi:10.3390/molecules24061060)

Supplementary Materials:

Table 2. Spectral data of 2-substituted 4*H*-benzo[*h*]chromene derivatives (4-11, 13, 14)

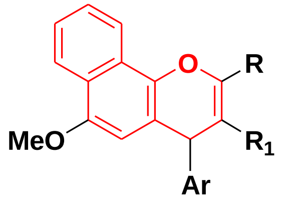

(4, 7-11)

Ar = 2,4-diMeOC$_6$H$_4$

4: R = NH$_2$, R$_1$ = CN
7: R = NHAc, R$_1$ = CN
8: R = N=CHPh, R$_1$ = CN
9: R = N=CHOEt, R$_1$ = CN
10: R = N=CHNMe$_2$, R$_1$ = CN
11: R = N=CHNH$_2$, R$_1$ = CN

Compound	Spectral data
**4**	IR (KBr, *υ* cm^{-1}): 3481, 3436, 3332 (NH_2), 3079, 3001 (CH-arom.), 2936, 2837 (CH-aliph.), 2186 (CN).<br>^{1}H-NMR (500 MHz, DMSO-d_6, *δ*, ppm); 3.73 (s, 3H, OCH_3), 3.82 (s, 3H, OCH_3), 3.86 (s, 3H, OCH_3), 5.13 (s, 1H, H-4), 6.97 (bs, 2H, NH_2, canceled by D_2O), 6.47-8.19 (m, 8H, Ar-H).<br>^{13}C NMR (500 MHz, DMSO-d_6, *δ*, ppm); 40.04 (C-4), 55.11 (CH_3), 55.19 (CH_3), 55.50 (CH_3), 55.68 (C-3), 98.67 (Ar-C), 102.96 (Ar-C), 105.37 (C-5), 118.33 (CN), 120.72 (C-4a), 120.49 (Ar-C), 121.53 (C-10), 123.58 (C-7), 124.18 (C-8), 125.57 (C-6a), 125.94 (C-9), 127.09 (C-10a), 129.28 (Ar-C), 136.93 (C-10b), 151.01 (C-6), 157.22 (Ar-C), 159.40 (Ar-C), 161.01 (C-2).<br>MS (*m/z*), 388 (M^+, 30.38) with a base peak at 374 (100).
**7**	IR (KBr, *υ* cm $^{-1}$): 3223 (NH), 3007, 3035 (CH-arom.), 2932, 2839, 2830(CH-aliph.), 2219 (CN), 1740 (CO).<br>^{1}H-NMR (500 MHz, DMSO-d_6, *δ*, ppm); 2.15 (s, 3H, $COCH_3$), 3.75 (s, 3H, OCH_3), 3.77 (s, 3H, OCH_3), 3.86 (s, 3H, OCH_3), 5.56 (s, 1H, H-4), 6.51-8.12 (m, 8H, Ar-H), 10.68 (s, 1H, NH).<br>^{13}C NMR (500 MHz, DMSO-d_6, *δ*, ppm); 24.50 (CH_3), 39.06(C-4), 55.16 (CH_3), 55.54 (CH_3), 55.68 (CH_3), 91.57 (C-3), 98.85 (Ar-C), 102.34 (C-5), 105.51 (Ar-C), 115.59 (Ar-C), 116.46 (CN), 120.43 (C-4a), 121.64 (C-10), 122.13 (C-7), 123.44 (C-6a), 124.46 (C-8), 126.47 (C-9), 127.74 (C-10a), 130.57 (Ar-C), 136.32 (C-10b), 152.26 (C-6), 152.37 (Ar-C), 157.41 (Ar-C), 160.30 (C-2), 170.64 (CO).<br>MS (m/z), 430 (M+, 34.28) with a base peak at 388 (100).
**8**	IR (KBr, *υ* cm^{-1}): 3063, 3001 (CH-arom.), 2940, 2836 (CH-aliph.), 2208 (CN). ^{1}H-NMR (500 MHz, DMSO-d_6, δ, ppm); 3.76 (s, 3H, OCH_3), 3.83 (s, 3H, OCH_3), 3.85(s, 3H, OCH_3), 5.45 (s, 1H, H-4), 6.51-8.48 (m, 13H, Ar-H), 9.35 (s, 1H, N=CH).<br>^{13}C NMR (500 MHz, DMSO-d_6, *δ*, ppm); 40.01 (C-4), 55.52 (CH_3), 55.62 (CH_3), 55.85 (CH_3), 86.62 (C-3), 98.94 (Ar-C), 102.99 (C-5), 105.66 (Ar-C), 116.63 (CN), 117.72 (Ar-C), 121.10 (C-4a), 121.57 (C-10), 123.97 (C-7), 124.55 (C-8), 126.35 (C-6a), 127.09 (C-9), 127.53 (C-10a),

	129.28 (Ar-C), 130.01 (Ar-C), 133.30 (Ar-C), 134.89 (Ar-C), 137.02 (C-10b), 151.76 (C-6), 157.61 (N=CH), 158.20 (Ar-C), 160.08(Ar-C), 162.72 (C-2).<br><br>MS (*m/z*), 476 (M^+, 13.91) with a base peak at 446 (100).
**9**	IR (KBr, υ cm^{-1}): 3079, 3008 (CH-arom.), 2994, 2937, 2840 (CH-aliph.), 2209 (CN).<br><br>^{1}H-NMR (500 MHz, DMSO-d_6, δ, ppm); 1.45 (t, 3H, CH_3, *J*=7.2 Hz), 3.80 (s, 3H, OCH_3), 3.87 (s, 3H, OCH_3), 3.91 (s, 3H, OCH_3), 4.50 (q, 2H, CH_2, *J*=7.2 Hz), 5.44 (s, 1H, H-4), 6.42-8.21 (m, 8H, Ar-H), 8.59 (s, 1H, N=CH).<br><br>^{13}C NMR (500 MHz, DMSO-d_6, δ, ppm); 13.89 (CH_3), 36.28 (C-4), 55.56 (CH_3), 55.80 (CH_3), 55.99 (CH_3), 64.27 (CH_2), 80.72 (C-3), 99.05 (Ar-C), 103.12 (Ar-C), 105.42 (C-5), 117.22 (CN), 118.72 (Ar-C), 120.55 (C-4a), 122.40 (C-10), 124.64 (C-7), 124.80 (C-8), 125.53 (C-6a), 126.08 (C-9), 127.28 (C-10a), 130.75 (Ar-C), 138.08 (C-10b), 152.53 (C-6), 157.91 (C-2), 157.93 (N=CH), 158.93 (Ar-C), 160.35 (Ar-C).<br><br>MS (*m/z*), 444 (M^+, 4.76) with a base peak at 430 (100).
**10**	IR (KBr, υ cm^{-1}): 3069, 3007(CH-arom.),2995, 2962, 2935, 2838 (CH-aliph.), 2195 (CN).<br><br>^{1}H-NMR (500 MHz, DMSO-d_6, δ, ppm); 3.25, 3.06 (s, 6H, $2CH_3$), 3.82 (s, 3H, OCH_3), 3.86 (s, 3H, OCH_3), 3.87 (s, 3H, OCH_3), 5.14 (s, 1H, H-4), 6.46-8.08 (m, 8H, Ar-H), 8.60 (s, 1H, N=CH).<br><br>^{13}C NMR (500 MHz, DMSO-d_6, δ, ppm); 34.16 (CH_3), 39.90 (C-4), 54.98 (CH_3), 55.08 (CH_3), 55.36 (CH_3), 98.54 (C-3), 102.82 (C-5), 105.24 (Ar-C), 118.20 (CN), 120.37 (Ar-C), 120.60 (C-4a), 121.40 (C-10), 123.46 (C-7), 124.06 (C-8), 125.45 (C-6a), 125.80 (C-9), 126.95 (C-10a), 129.16 (Ar-C), 136.80 (C-10b), 150.88 (C-6), 157.10 (C-2), 159.27 (Ar-C), 159.45 (N=CH), 160.89 (Ar-C).<br><br>MS (*m/z*), 443 (M^+, 7.09) with a base peak at 99 (100).
**11**	IR (KBr, υ cm^{-1}): 3472, 3412 (NH_2), 3014, 3003 (CH-arom.), 2995, 2980, 2937, 2840 (CH-aliph.), 2208 (CN).<br><br>^{1}H-NMR (500 MHz, DMSO-d_6, δ, ppm); 3.74 (s, 3H, OCH_3), 3.82 (s, 3H, OCH_3), 3.87 (s, 3H, OCH_3), 5.26 (s, 1H, H-4), 7.69 (s, 2H, NH_2), 8.10 (s, 1H, N=CH), 6.47-8.59 (m, 8H, Ar-H).<br><br>^{13}C NMR (500 MHz, DMSO-d_6, δ, ppm); 35.63 (C-4), 55.13 (CH_3), 55.50 (CH_3), 55.75 (CH_3), 71.49 (C-3), 98.69 (Ar-C), 102.86 (C-5), 105.52 (Ar-C), 117.68 (CN), 120.27 (Ar-C), 120.58 (C-4a), 121.56 (C-10), 123.93 (C-7), 124.31 (C-8), 125.02 (C-6a), 125.97 (C-9), 127.19 (C-10a), 129.68 (Ar-C), 137.24 (C-10b), 151.09 (C-6), 154.53 (Ar-C), 157.23 (Ar-C), 159.53 (C-2), 160.97 (N=CH).<br><br>MS (*m/z*), 415 (M^+, 6.63) with a base peak at 79 (100).

Table 3. Spectral data of 7*H*-benzo[*h*]chromeno[2,3-*d*]pyrimidine derivatives (12-16).

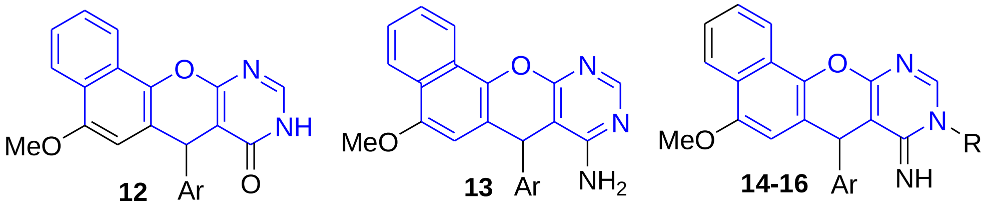

Ar = 2,4-diMeOC_6H_4

14: R = Me
15: R = NH_2
16: R = N=CHPh

Compound	Spectral data
**12**	IR (KBr, υ cm^{-1}): 3487 (NH), 3076, 3015 (CH-arom.), 2969, 2942, 2876, 2835 (CH-aliph.), 1757 (CO).<br>^{1}H-NMR (500 MHz, DMSO-d_6, δ, ppm); 3.75 (s, 3H, OCH_3), 3.83 (s, 3H, OCH_3), 3.89 (s, 3H, OCH_3), 5.30 (s, 1H, H-7), 8.13 (bs, 1H, NH), 6.49-8.15 (m, 10H, Ar-H, H-10).<br>^{13}C NMR (500 MHz, DMSO-d_6, δ, ppm); 38.06 (C-7), 55.21 (CH_3), 55.62 (CH_3), 55.88 (CH_3), 98.82 (Ar-C), 103.46 (C-7a), 105.61 (Ar-C), 115.30 (Ar-C), 118.50 (C-6), 120.34 (C-6a), 121.71 (C-1), 124.66 (C-4), 126.48 (C-3), 127.65 (C-2), 127.73 (C-4a), 128.29 (C-1a), 130.48 (Ar-C), 138.49 (C-1b), 151.46 (C-10), 151.50 (C-5), 157.89 (Ar-C), 158.70 (Ar-C), 160.70 (C-11a), 161.41 (CO).<br>MS (*m/z*), 416 (M^+, 24.92) with a base peak at 292 (100)
**13**	IR (KBr, υ cm^{-1}): 3492, 3385, 3221 (NH_2), 3012(CH-arom.), 2969, 2941, 2841 (CH-aliph.).<br>^{1}H-NMR (500 MHz, DMSO-d_6, δ, ppm); 3.71 (s, 3H, OCH_3), 3.81 (s, 3H, OCH_3), 3.85 (s, 3H, OCH_3), 5.45 (s, 1H, H-7), 6.56 (s, 2H, NH_2), 8.10 (s, 1H, H-10), 6.44-8.26 (m, 8H, Ar-H).<br>^{13}C NMR (500 MHz, DMSO-d_6, δ, ppm); 32.92 (C-7), 55.16 (CH_3), 55.62 (CH_3), 55.88 (CH_3), 95.36 (Ar-C), 98.98 (C-7a), 105.87 (C-6), 103.42 (Ar-C), 118.59 (C-6a), 120.67 (Ar-C), 121.59 (C-1), 123.72 (C-4), 124.05 (C-3), 124.41 (C-4a), 126.08 (C-2), 127.31 (C-1a), 130.10 (Ar-C), 138.16 (C-1b), 151.04 (C-5), 156.16 (Ar-C), 157.00 (Ar-C), 159.62 (C-10), 162.45 (C-11a), 162.53 (C-8).<br>MS (*m/z*), 415 (M^+, 84.18) with a base peak at 374 (100).
**14**	IR (KBr, υ cm^{-1}): 3327 (NH), 3077, 3029 (CH-arom.), 2989, 2934, 2835 (CH-aliph.).<br>^{1}H-NMR (500 MHz, DMSO-d_6, δ, ppm); 3.31 (s, 3H, CH_3), 3.70 (s, 3H, OCH_3), 3.82 (s, 3H, OCH_3), 3.95 (s, 3H, OCH_3), 5.38 (s, 1H, H-7), 6.52 (bs, 1H, NH), 8.14 (s, 1H, H-10), 6.40-8.19 (m, 8H, Ar-H).<br>^{13}C NMR (500 MHz, DMSO-d_6, δ, ppm); 32.24 (CH_3), 35.24 (C-7), 55.11 (CH_3), 55.45 (CH_3), 55.98 (CH_3), 97.41 (C-7a), 98.43 (Ar-C), 97.41 (C-7a), 103.07 (C-6), 106.05 (Ar-C), 118.37 (Ar-C),120.60 (C-1), 121.53 (C-4), 123.92 (C-6a), 124.30 (C-1a, 4a), 125.96 (C-3), 127.24 (C-2), 129.66 (Ar-C), 137.26 (C-1b), 150.83 (C-10), 151.19 (C-5,11a), 156.75 (Ar-C), 159.41 (C-8).<br>^{13}C NMR-DEPT 135° CH, CH_3 (↑), CH_2 (↓) (125 MHz, $CDCl_3$, δ, ppm); 32.24 (CH_3↑),35.24 (C-7 ↑), 55.11 (CH_3↑), 55.45 (CH_3↑), 55.98 (CH_3↑), 98.43 (Ar-CH↑),103.07 (C-6 ↑), 106.05 (Ar-CH

↑), 120.60 (C-1 ↑), 121.53 (C-4 ↑), 125.96 (C-3 ↑), 127.24 (C-2 ↑), 129.66 (Ar-CH ↑), 150.83 (C-10 ↑).

^{13}C NMR-DEPT 90º CH (↑) (125 MHz, $CDCl_3$, δ, ppm); 35.24 (C-7 ↑), 98.43 (Ar-CH ↑),103.07 (C-6 ↑), 106.05 (Ar-CH ↑), 120.60 (C-1 ↑), 121.53 (C-4 ↑), 125.96 (C-3 ↑), 127.24 (C-2 ↑), 129.66 (Ar-CH ↑), 150.83 (C-10 ↑).

^{13}C NMR-DEPT 45º CH, CH_2, CH_3 (↑) (125 MHz, $CDCl_3$, δ, ppm); 32.24 (CH_3↑), 35.24 (C-7 ↑), 55.11 (CH_3↑), 55.45 (CH_3↑), 55.98 (CH_3↑), 98.43 (Ar-CH ↑), 103.07 (C-6 ↑), 106.05 (Ar-CH ↑), 120.60 (C-1 ↑), 121.53 (C-4 ↑), 125.96 (C-3 ↑), 127.24 (C-2 ↑), 129.66 (Ar-CH ↑), 150.83 (C-10 ↑).

^{13}CNMR-APT spectrum CH, CH_3 [positive (up)], CH_2, Cq [negative (down)], revealed the following signals (125 MHz, $CDCl_3$, δ, ppm); 32.24 (CH_3↑), 35.24 (C-7 ↑), 55.11 (CH_3↑),55.45 (CH_3↑), 55.98 (CH_3↑), 97.41 (C-7a↓), 98.43 (Ar-CH ↑), 103.07 (C-6 ↑), 106.05 (Ar-CH ↑), 118.37 (Ar-C ↓), 120.60 (C-1 ↑), 121.53 (C-4 ↑), 123.92 (C-6a ↓), 124.30 (C-1a, 4a ↓), 125.96 (C-3 ↑), 127.24 (C-2 ↑), 129.66 (Ar-CH ↑), 137.26 (C-1b ↓), 150.83 (C-10 ↑), 151.19 (C-5, 11a ↓), 156.75 (Ar-C ↓), 159.41 (C-8 ↓).

MS (*m*/*z*), 429 (M^+, 50.8) with a base peak at 292 (100).

15 IR (KBr, υ cm^{-1}): 3334, 3297, 3136 (NH & NH_2), 3068, 2939, 2831 (CH-aliph.). ^{1}H-NMR (500 MHz, DMSO-d_6, δ, ppm); 3.70 (s, 3H, OCH_3), 3.84 (s, 3H, OCH_3), 3.93 (s, 3H, OCH_3), 5.44 (s, 1H, H-7), 5.69 (bs, 2H, NH_2), 6.61 (bs, 1H, NH), 8.14 (s, 1H, H-10), 6.41-8.20 (m, 8H, Ar-H).

^{13}C NMR (500 MHz, DMSO-d_6, δ, ppm); 39.92 (C-7), 55.12 (CH_3), 55.47 (CH_3), 55.94 (CH_3), 97.51 (C-7a), 98.51 (Ar-C), 103.10 (C-6), 106.00 (Ar-C), 118.10 (Ar-C), 120.58 (C-6a), 121.54 (C-1), 123.91 (C-4), 124.28 (C-3), 125.96 (C-4a), 127.24 (C-2), 128.29 (C-1a), 130.00 (Ar-C), 137.40 (C-1b), 142.50 (C-10), 150.10 (C-5), 151.29 (C-11a), 156.14 (Ar-C), 156.89 (Ar-C), 159.41 (C-8).

MS (*m*/*z*), 430 (M^+, 12.34) with a base peak at 416 (100).

16 IR (KBr, υ cm^{-1}): 3283 (NH), 3026, 3000 (CH-arom.),2968, 2899, 2840 (CH-aliph.), 1644 (C=N).

^{1}H-NMR (500 MHz, DMSO-d_6, δ, ppm); 3.65 (s, 3H, OCH_3), 3.77 (s, 3H, OCH_3), 3.94 (s, 3H, OCH_3), 6.50 (s, 1H, H-7), 8.11 (s, 1H, H-10), 6.36-8.27 (m, 13H, aromatic), 8.42 (s, 1H, N=CH), 10.70 (bs, 1H, NH).

^{13}C NMR (500 MHz, DMSO-d_6, δ, ppm); 33.42 (C-7), 55.05 (CH_3), 55.36 (CH_3), 55.63 (CH_3), 96.81 (C-7a), 98,81 (Ar-C), 103.49 (C-6), 105,19 (Ar-C), 119.34 (Ar-C), 120.48 (C-6a), 123.92 (C-1), 124.30 (C-4), 124.44 (C-3), 125.97 (C-4a), 126.78 (C-2), 127.27 (C-1a), 128.26 (Ar-C), 128.34 (Ar-C), 129.02 (Ar-C), 129.49 (Ar-C), 134.33 (Ar-C), 137.79 (C-1b), 143.95 (C-5), 151.13 (C-11a), 156.12 (N=CH), 156.97 (Ar-C), 158.32 (Ar-C), 159.52 (C-10), 164.65 (C-8).

MS (*m*/*z*), 518 (M^+, 1.24) with a base peak at 77 (100).

NMR Spectra of Compounds 4, 7-11, 12, 13 and 14-16

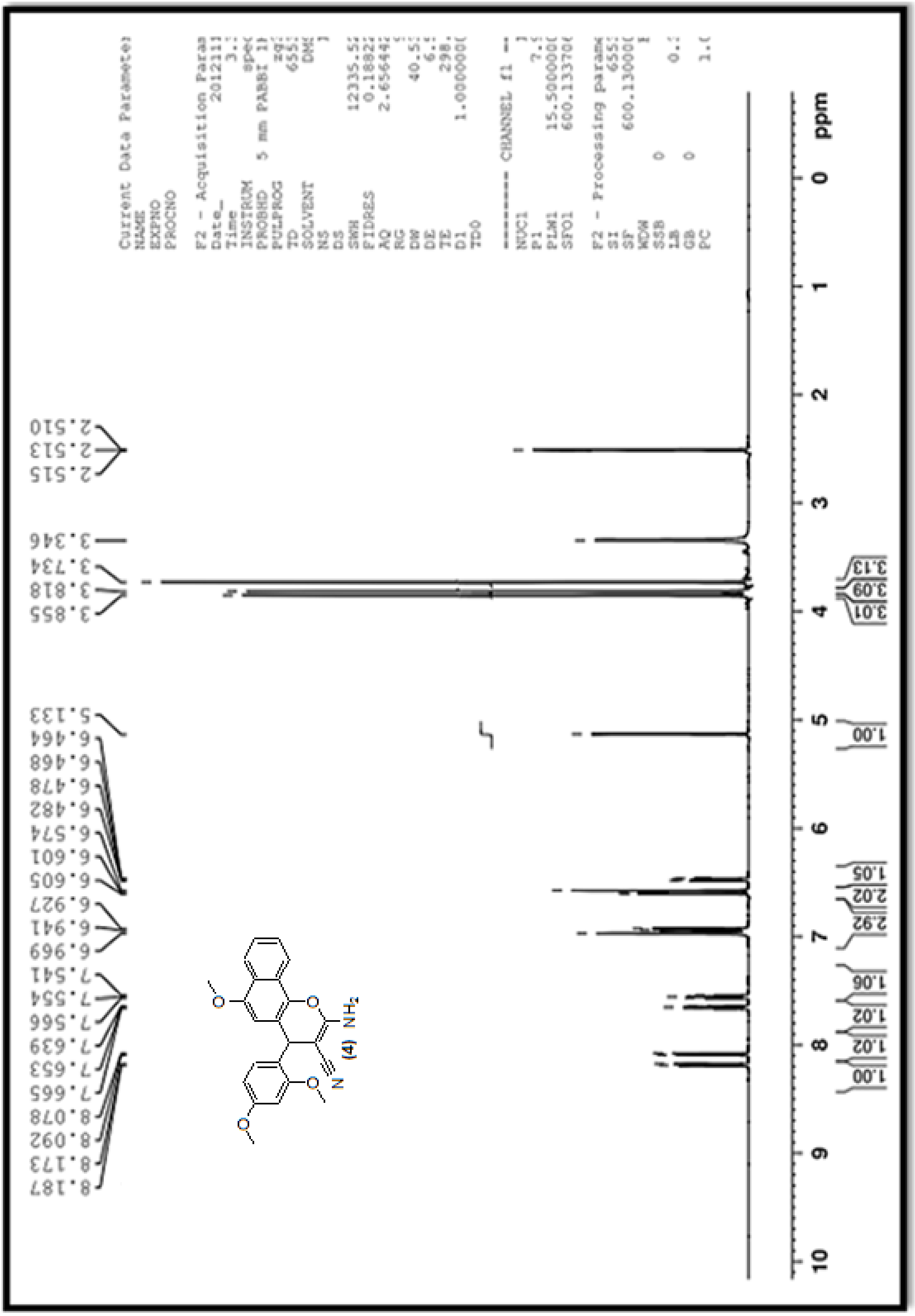

105 e

NMR 500 MHz Ultra Shield™

BRUKER

1H (AG-5M)

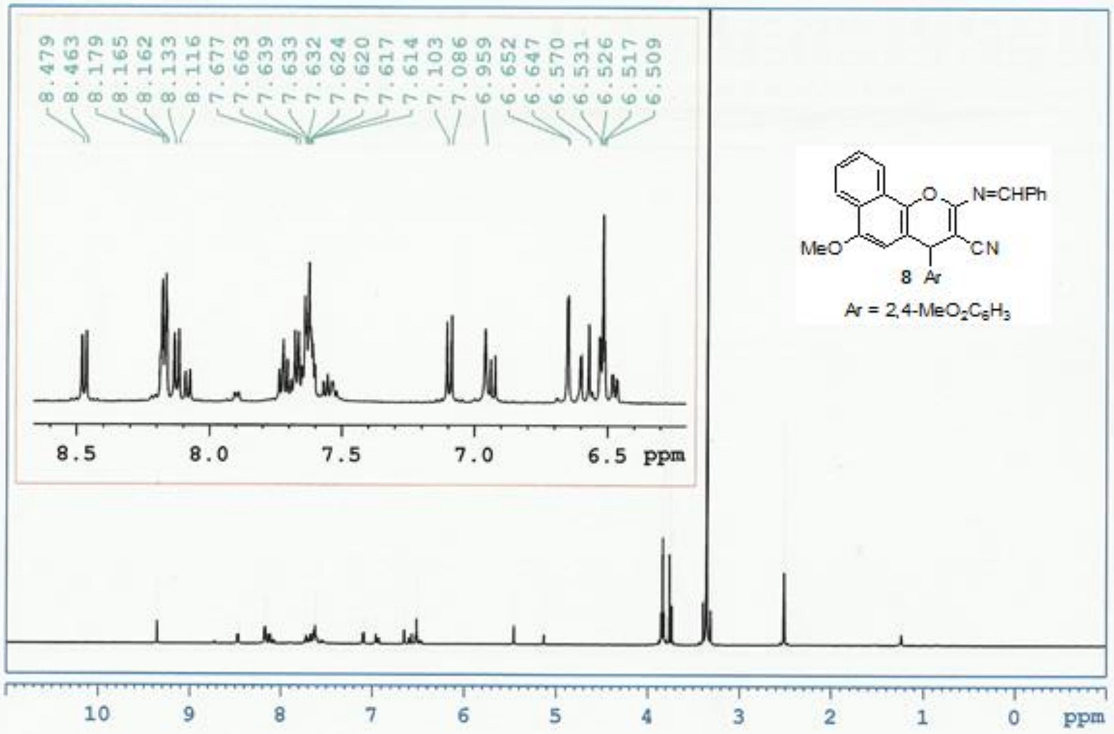

NAME Apr11-2013-nmr
EXPNO 140
PROCNO 1
Date_ 20130415
Time 19.52
INSTRUM spect
PROBHD 5 mm PABBO BB-
PULPROG zg30
TD 65536
SOLVENT DMSO
NS 64
DS 2
SWH 10330.578 Hz
FIDRES 0.157632 Hz
AQ 3.1719923 sec
RG 203
DW 48.400 usec
DE 6.50 usec
TE 268.5 K
D1 1.00000000 sec
TD0 1

======== CHANNEL f1 ========
NUC1 1H
P1 14.80 usec
PL1 3.40 dB
PL1W 12.17042828 W
SFO1 500.1330885 MHz
SI 32768
SF 500.1300000 MHz
WDW EM
SSB 0
LB 0.30 Hz
GB 0
PC 1.00

NMR 500 MHz Ultra Shield™

1H (AG-5M)

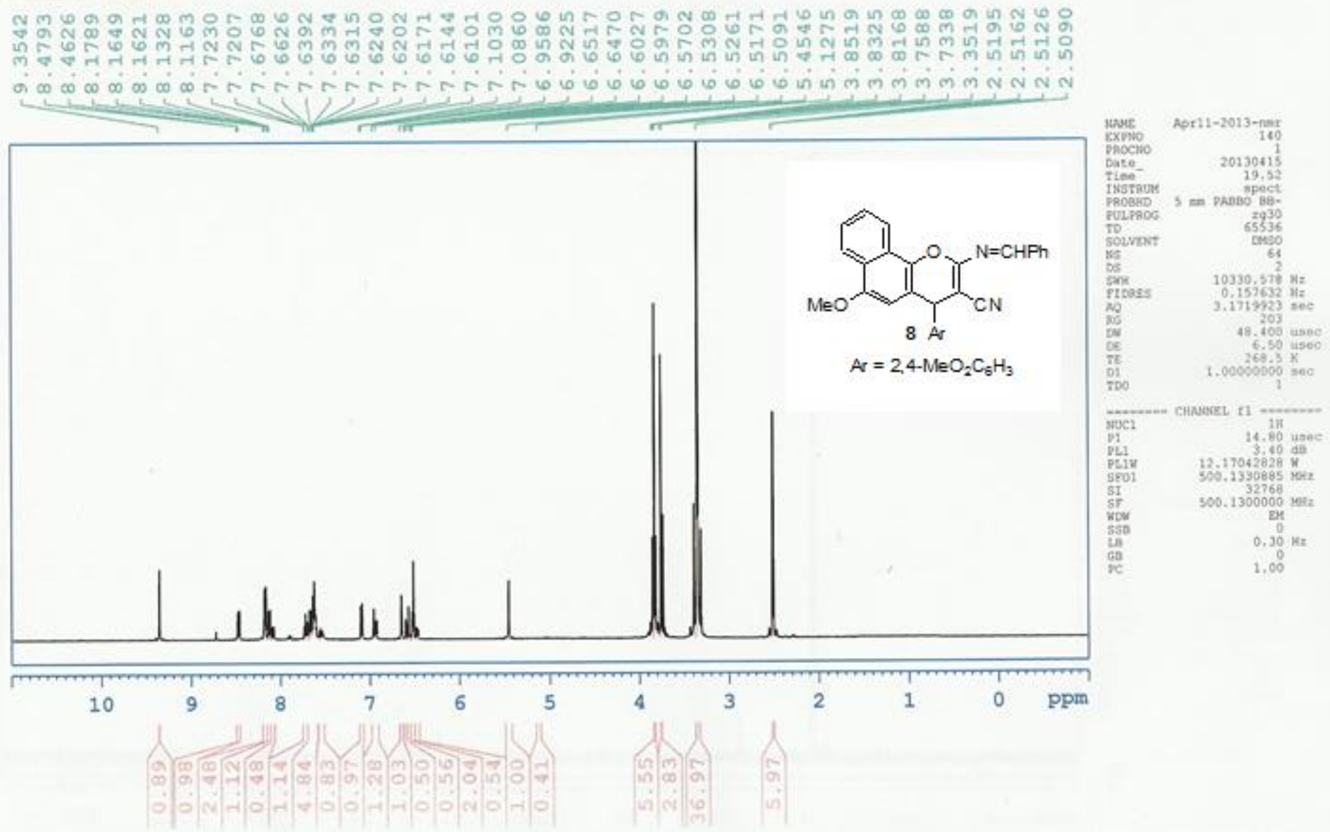

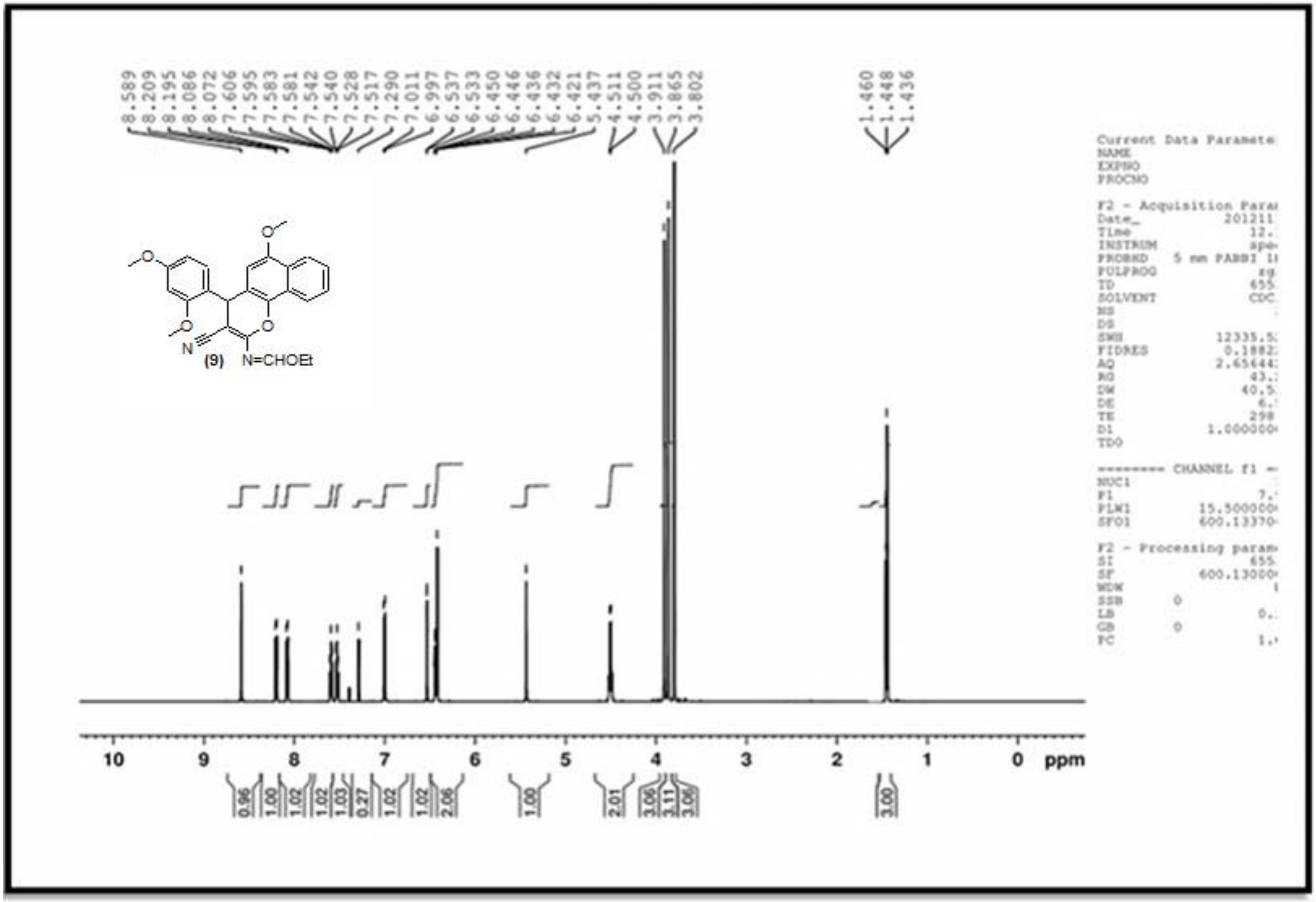

8.589 8.209 8.195 8.086 8.072 7.606 7.595 7.583 7.581 7.542 7.540 7.528 7.517 7.290 7.011 6.997 6.537 6.533 6.450 6.446 6.436 6.432 6.421 5.437 4.511 4.500 3.911 3.865 3.802
1.460 1.448 1.436
(9)
N=CHOEt
10 9 8 7 6 5 4 3 2 1 0 ppm
0.96 1.00 1.02 1.02 1.03 0.27 1.02 1.02 2.06 1.00 2.01 3.06 3.11 3.06 3.00
Current Data Paramete
NAME
EXPNO
PROCNO
F2 - Acquisition Para
Date_ 201211
Time 12.
INSTRUM spe
PROBHD 5 mm PABBI 1
PULPROG zg
TD 655
SOLVENT CDC
NS
DS
SWH 12335.5
FIDRES 0.1882
AQ 2.65644
RG 43.
DW 40.5
DE 6.
TE 298
D1 1.000000
TD0
======== CHANNEL f1 =
NUC1
P1 7.
PLW1 15.500000
SFO1 600.13370
F2 - Processing param
SI 655
SF 600.13000
WDW
SSB 0
LB 0.
GB 0
PC 1.

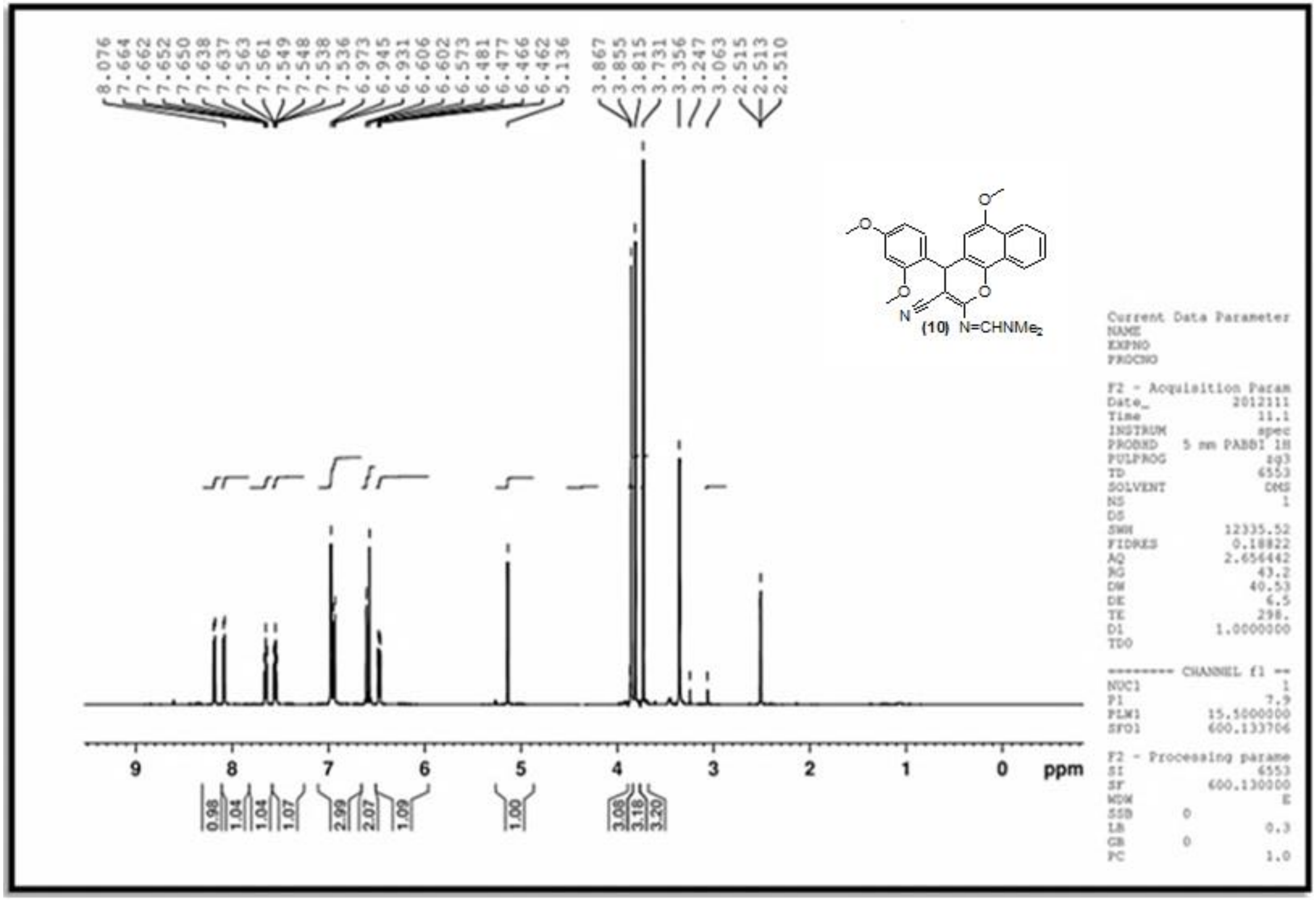

8.076 7.664 7.662 7.652 7.650 7.638 7.637 7.563 7.561 7.549 7.548 7.538 7.536 6.973 6.945 6.931 6.606 6.602 6.573 6.481 6.477 6.466 6.462 5.136
3.867 3.855 3.815 3.731 3.356 3.247 3.063 2.515 2.513 2.510
(10) N=CHNMe2
9 8 7 6 5 4 3 2 1 0 ppm
0.98 1.04 1.04 1.07 2.99 2.07 1.09 1.00 3.08 3.18 3.20
Current Data Parameter
NAME
EXPNO
PROCNO
F2 - Acquisition Param
Date_ 2012111
Time 11.1
INSTRUM spec
PROBHD 5 mm PABBI 1H
PULPROG zg3
TD 6553
SOLVENT DMS
NS 1
DS
SWH 12335.52
FIDRES 0.18822
AQ 2.656442
RG 43.2
DW 40.53
DE 6.5
TE 298.
D1 1.0000000
TD0
======== CHANNEL f1 ==
NUC1 1
P1 7.9
PLW1 15.5000000
SFO1 600.133706
F2 - Processing parame
SI 6553
SF 600.130000
WDW E
SSB 0
LB 0.3
GB 0
PC 1.0

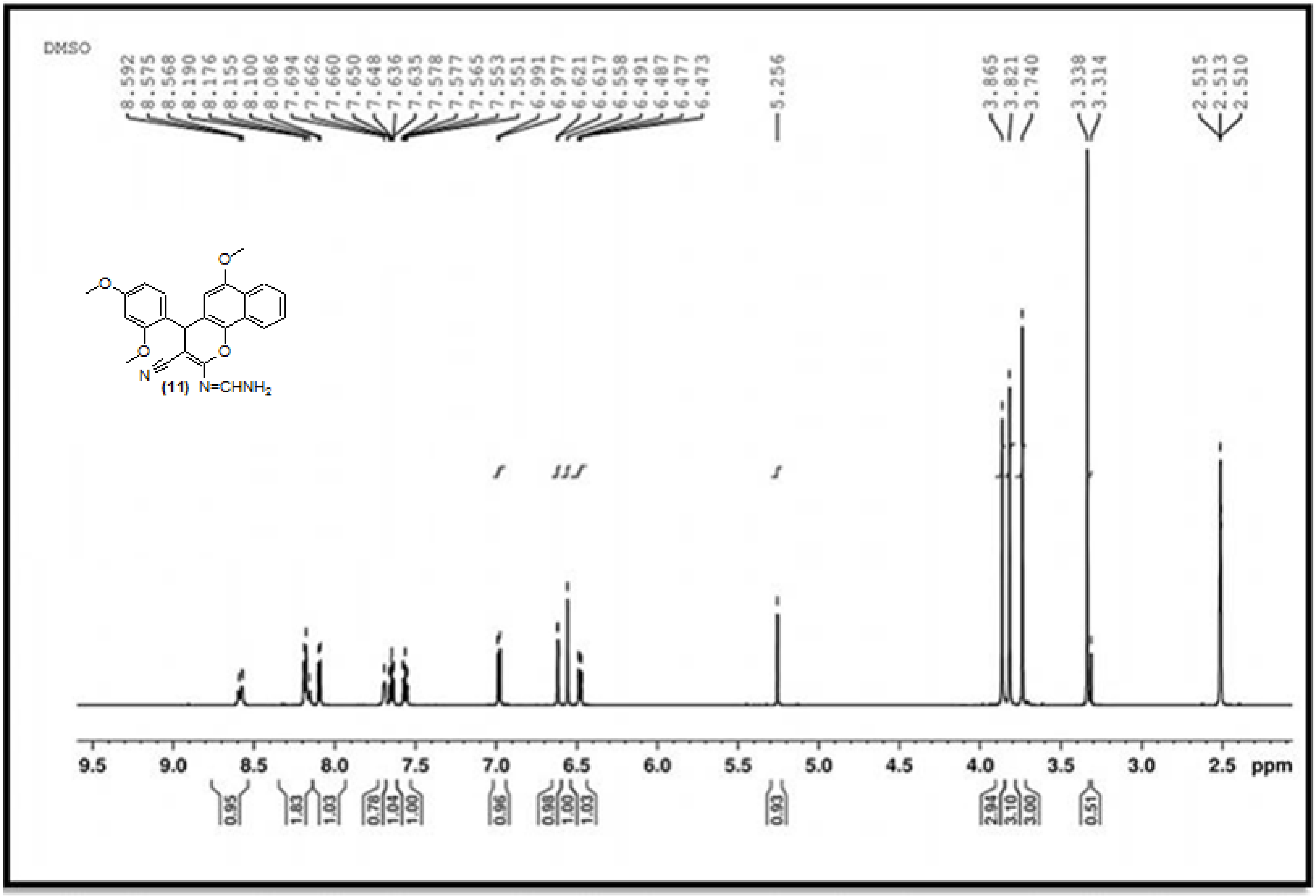
DMSO
8.592 8.575 8.568 8.190 8.176 8.155 8.100 8.086 7.694 7.662 7.660 7.650 7.648 7.636 7.635 7.578 7.577 7.565 7.553 7.551 6.991 6.977 6.621 6.617 6.558 6.491 6.487 6.477 6.473
5.256
3.865 3.821 3.740
3.338 3.314
2.515 2.513 2.510
(11) N=CHNH2
9.5 9.0 8.5 8.0 7.5 7.0 6.5 6.0 5.5 5.0 4.5 4.0 3.5 3.0 2.5 ppm
0.95 1.83 1.03 0.78 1.04 1.00 0.96 0.98 1.00 1.03 0.93 2.94 3.10 3.00 0.51

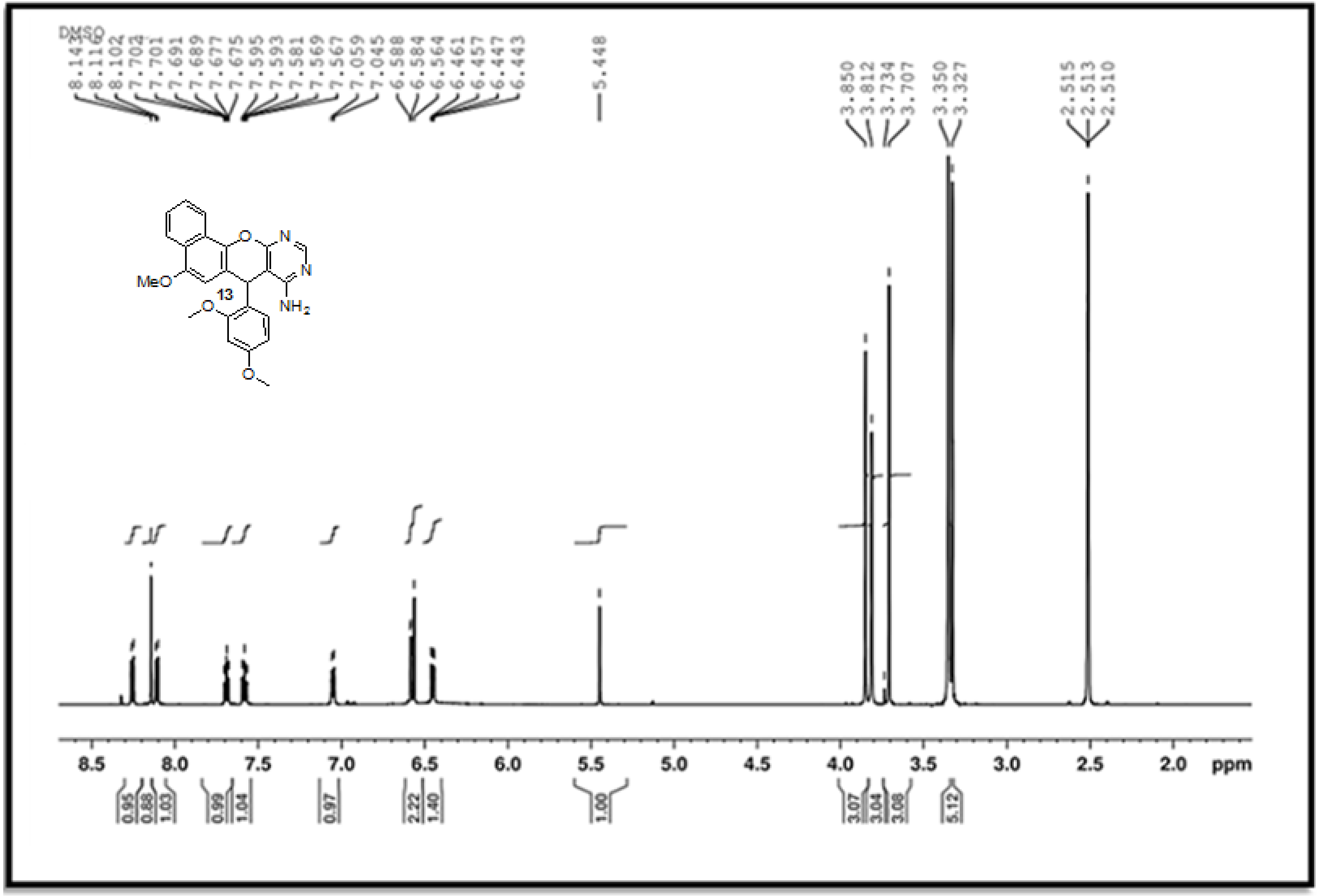
DMSO
8.143
8.116
8.102
7.703
7.701
7.691
7.689
7.677
7.675
7.595
7.593
7.581
7.569
7.567
7.059
7.045
6.588
6.584
6.564
6.461
6.457
6.447
6.443
5.448
3.850
3.812
3.734
3.707
3.350
3.327
2.515
2.513
2.510
MeO
13
NH2
8.5
8.0
7.5
7.0
6.5
6.0
5.5
5.0
4.5
4.0
3.5
3.0
2.5
2.0
ppm
0.95
0.88
1.03
0.99
1.04
0.97
2.22
1.40
1.00
3.07
3.04
3.08
5.12

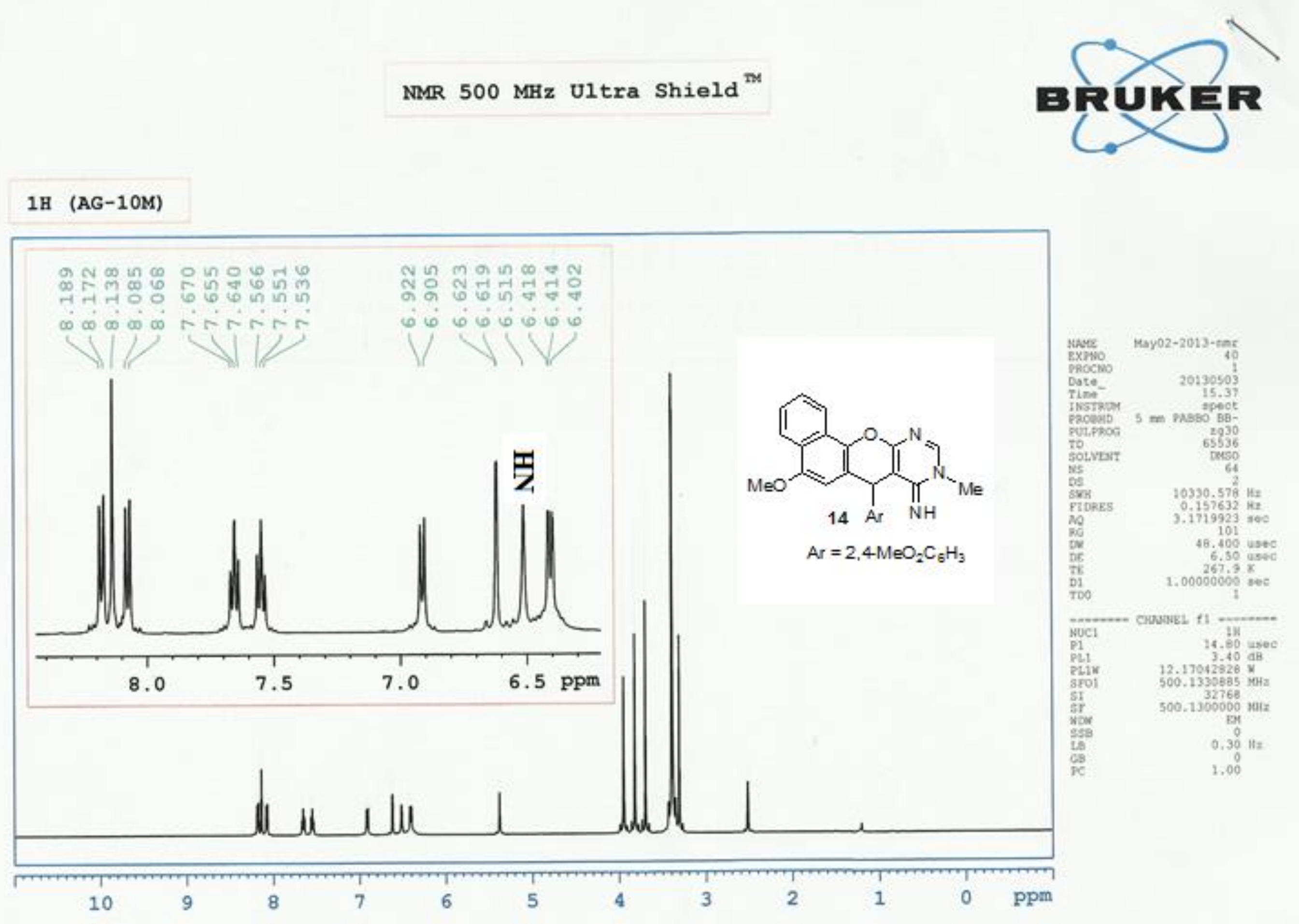

NMR 500 MHz Ultra Shield™
1H (AG-10M)
8.189
8.172
8.138
8.085
8.068
7.670
7.655
7.640
7.566
7.551
7.536
6.922
6.905
6.623
6.619
6.515
6.418
6.414
6.402
HN
8.0
7.5
7.0
6.5 ppm
MeO
N
N
Me
NH
14
Ar
Ar = 2,4-MeO2C6H3
NAME May02-2013-nmr
EXPNO 40
PROCNO 1
Date_ 20130503
Time 15.37
INSTRUM spect
PROBHD 5 mm PABBO BB-
PULPROG zg30
TD 65536
SOLVENT DMSO
NS 64
DS 2
SWH 10330.578 Hz
FIDRES 0.157632 Hz
AQ 3.1719923 sec
RG 101
DW 48.400 usec
DE 6.50 usec
TE 267.9 K
D1 1.00000000 sec
TD0 1
======== CHANNEL f1 ========
NUC1 1H
P1 14.80 usec
PL1 3.40 dB
PL1W 12.17042828 W
SFO1 500.1330885 MHz
SI 32768
SF 500.1300000 MHz
WDW EM
SSB 0
LB 0.30 Hz
GB 0
PC 1.00
10
9
8
7
6
5
4
3
2
1
0
ppm

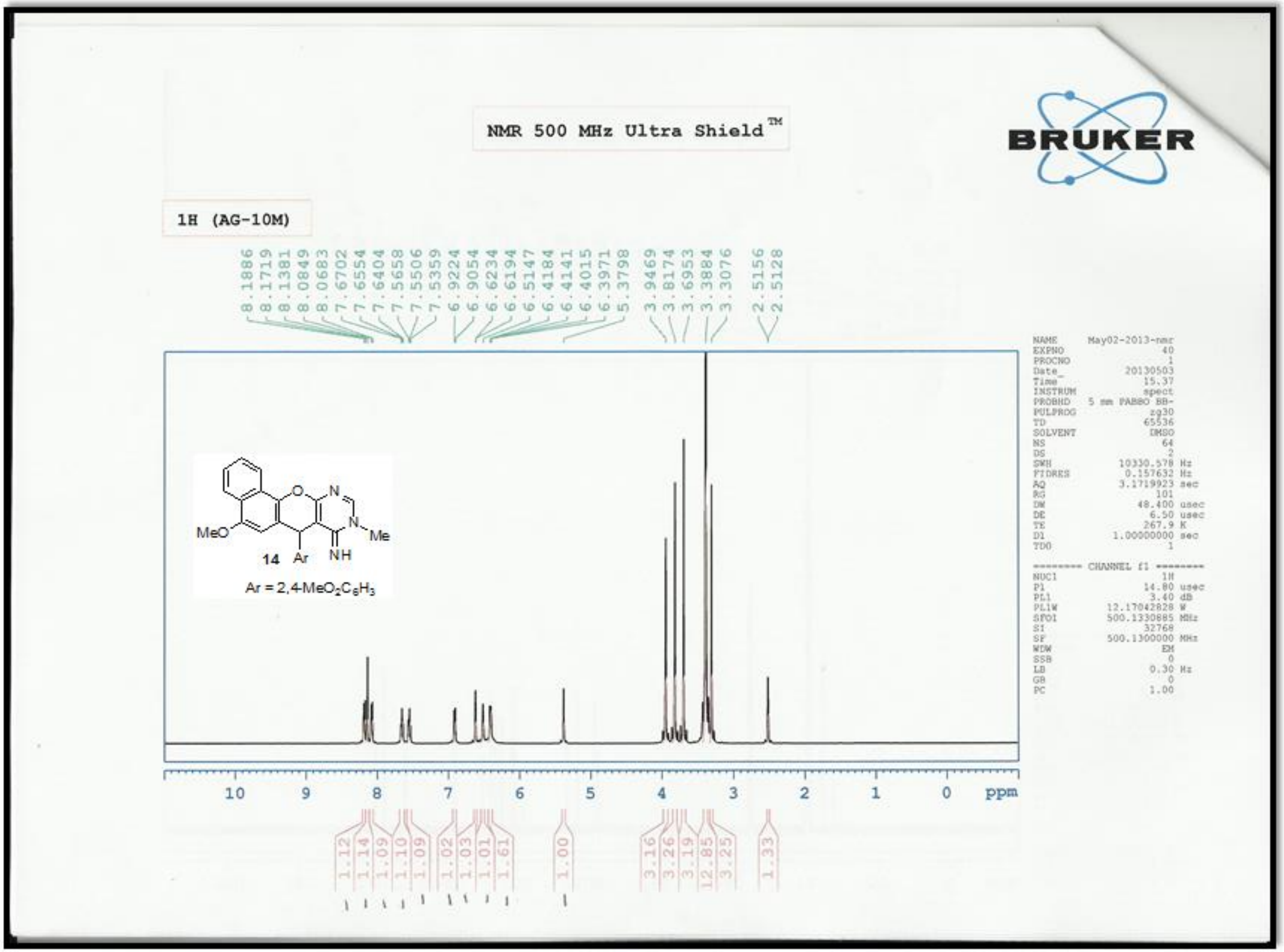
NMR 500 MHz Ultra Shield™
BRUKER
1H (AG-10M)
8.1886
8.1719
8.1381
8.0849
8.0683
7.6702
7.6554
7.6404
7.5658
7.5506
7.5359
6.9224
6.9054
6.6234
6.6194
6.5147
6.4184
6.4141
6.4015
6.3971
5.3798
3.9469
3.8174
3.6953
3.3884
3.3076
2.5156
2.5128
MeO
N
O
N
Me
NH
14
Ar
Ar = 2,4-MeO2C6H3
NAME May02-2013-nmr
EXPNO 40
PROCNO 1
Date_ 20130503
Time 15.37
INSTRUM spect
PROBHD 5 mm PABBO BB-
PULPROG zg30
TD 65536
SOLVENT DMSO
NS 64
DS 2
SWH 10330.578 Hz
FIDRES 0.157632 Hz
AQ 3.1719923 sec
RG 101
DW 48.400 usec
DE 6.50 usec
TE 267.9 K
D1 1.00000000 sec
TD0 1
======== CHANNEL f1 ========
NUC1 1H
P1 14.80 usec
PL1 3.40 dB
PL1W 12.17042828 W
SFO1 500.1330885 MHz
SI 32768
SF 500.1300000 MHz
WDW EM
SSB 0
LB 0.30 Hz
GB 0
PC 1.00
10
9
8
7
6
5
4
3
2
1
0
ppm
1.12
1.14
1.09
1.10
1.09
1.02
1.03
1.01
1.61
1.00
3.16
3.26
3.19
12.85
3.25
1.33

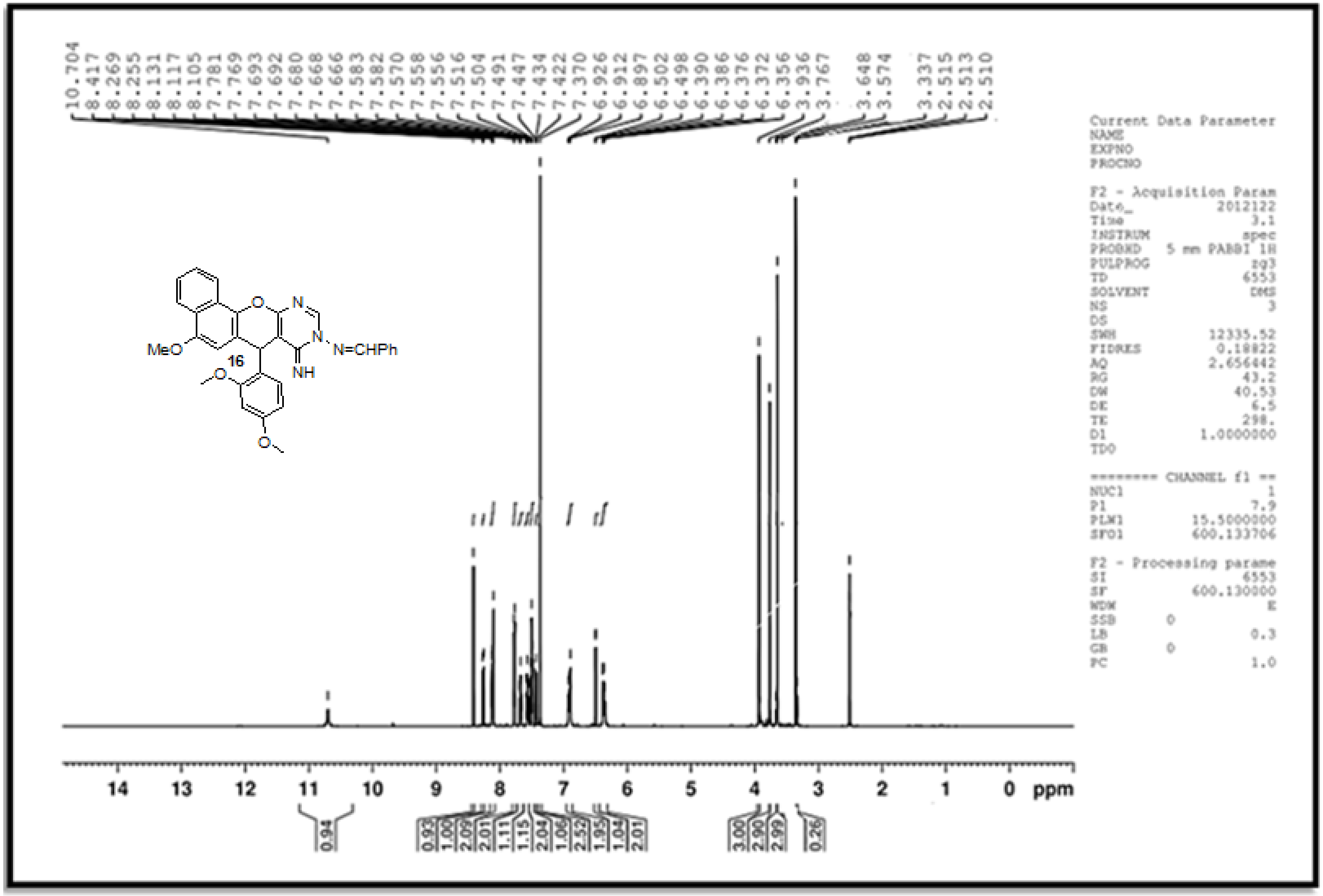
10.704
8.417
8.269
8.255
8.131
8.117
8.105
7.781
7.769
7.693
7.692
7.680
7.668
7.666
7.583
7.582
7.570
7.558
7.556
7.516
7.504
7.491
7.447
7.434
7.422
7.370
6.926
6.912
6.897
6.502
6.498
6.390
6.386
6.376
6.372
6.356
3.936
3.767
3.648
3.574
3.337
2.515
2.513
2.510
MeO
16
NH
N=CHPh
14 13 12 11 10 9 8 7 6 5 4 3 2 1 0 ppm
0.94
0.93
1.00
2.09
2.01
1.11
1.15
2.04
1.06
2.52
1.95
1.04
2.01
3.00
2.90
2.99
0.26
Current Data Parameter
NAME
EXPNO
PROCNO
F2 - Acquisition Param
Date_ 2012122
Time 3.1
INSTRUM spec
PROBHD 5 mm PABBI 1H
PULPROG zg3
TD 6553
SOLVENT DMS
NS 3
DS
SWH 12335.52
FIDRES 0.18822
AQ 2.656442
RG 43.2
DW 40.53
DE 6.5
TE 298.
D1 1.0000000
TD0
======== CHANNEL f1 ==
NUC1 1
P1 7.9
PLW1 15.5000000
SFO1 600.133706
F2 - Processing parame
SI 6553
SF 600.130000
WDW E
SSB 0
LB 0.3
GB 0
PC 1.0

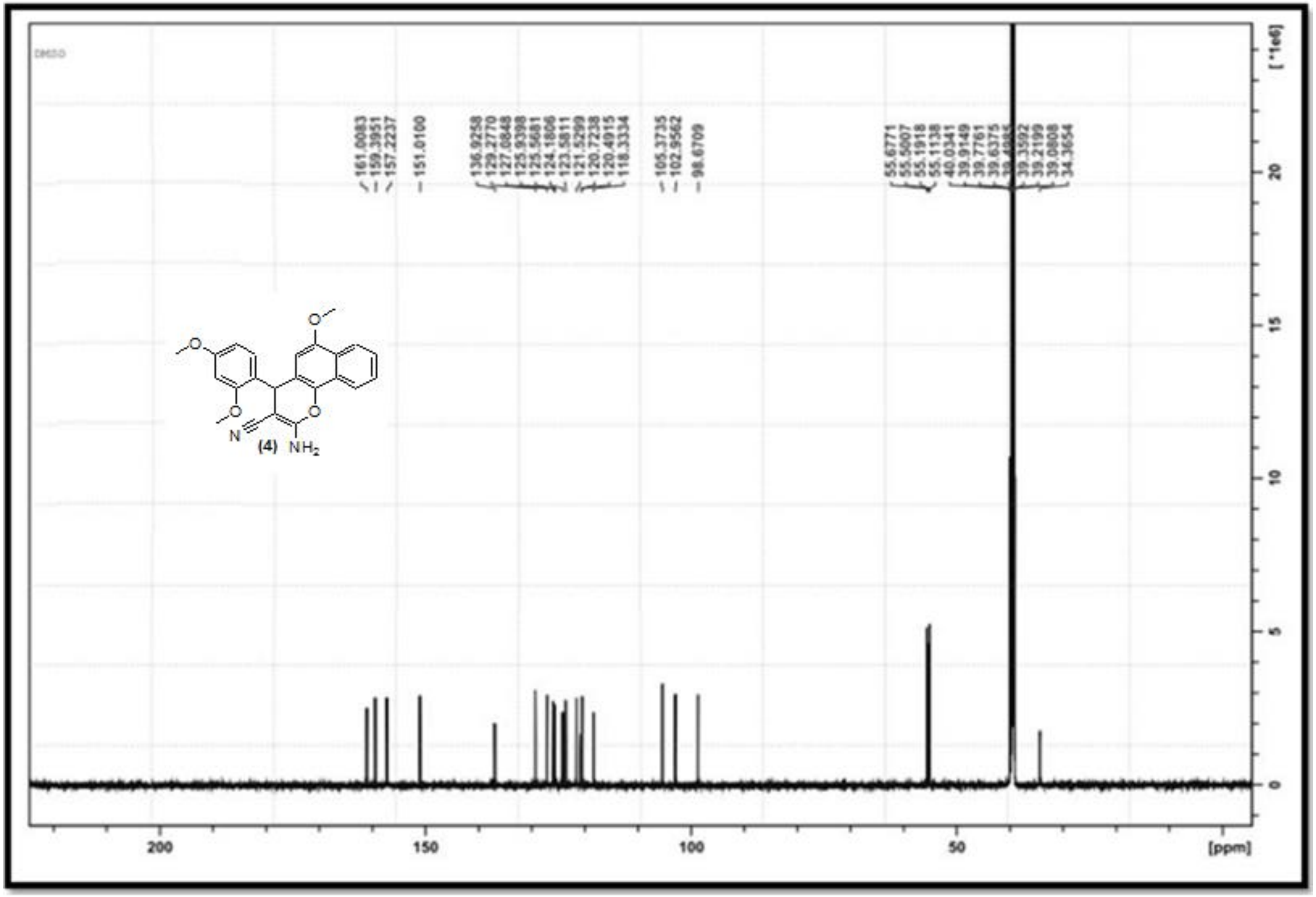
DMSO
161.0083
159.3951
157.2237
151.0100
136.9258
129.2770
127.0848
125.9398
125.5681
124.1806
123.5811
121.5299
120.7238
120.4915
118.3334
105.3735
102.9562
98.6709
55.6771
55.5007
55.1918
55.1138
40.0341
39.9149
39.7761
39.6375
39.4985
39.3592
39.2199
39.0808
34.3654
(4)
NH2
N
O
[ppm]
[*1e6]
200
150
100
50
0
5
10
15
20

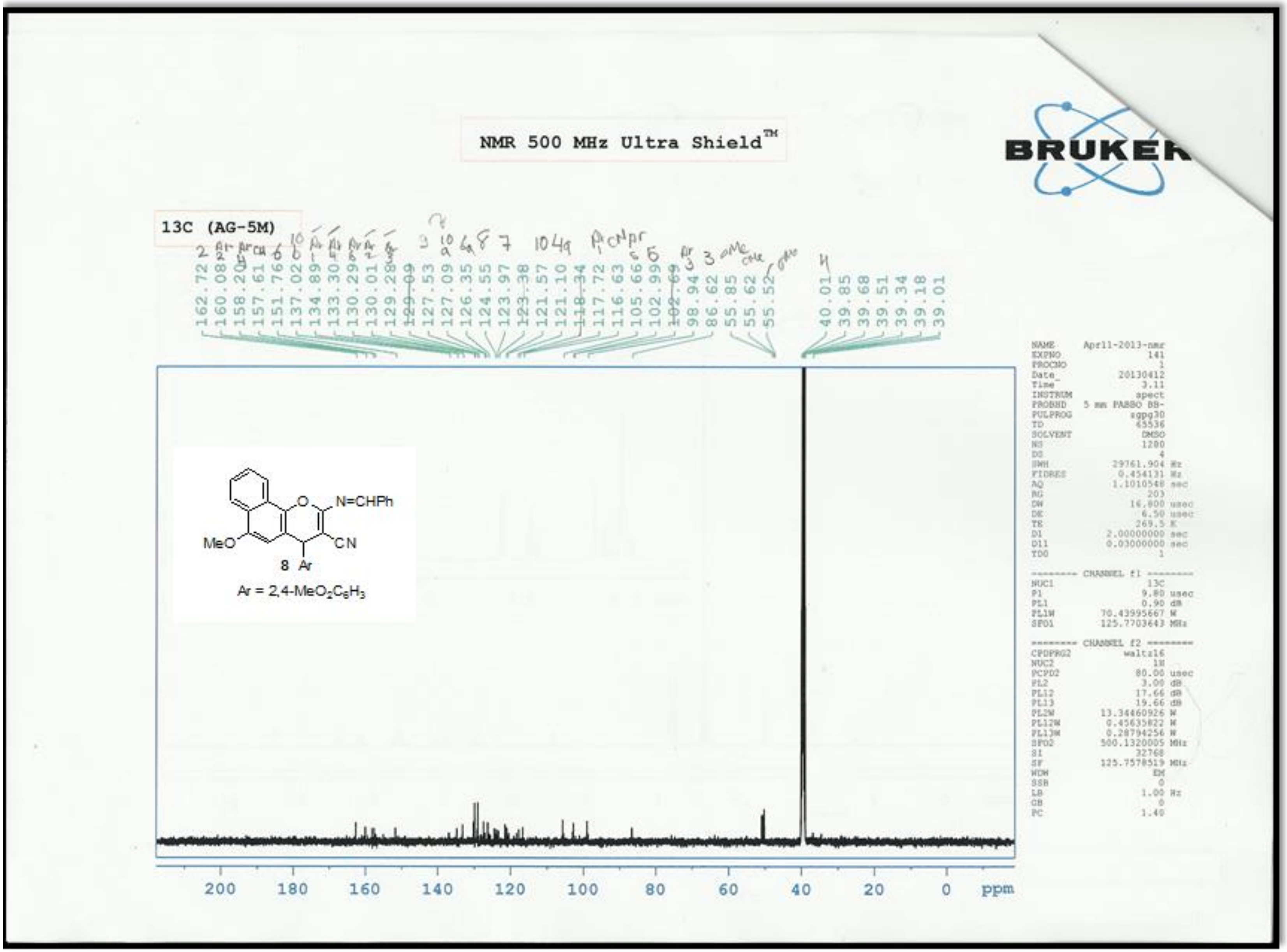
NMR 500 MHz Ultra Shield™
BRUKER
13C (AG-5M)
162.72
160.08
158.20
157.61
151.76
137.02
134.89
133.30
130.29
130.01
129.28
129.09
127.53
127.09
126.35
124.55
123.97
123.38
121.57
121.10
118.34
117.72
116.63
105.66
102.99
102.69
98.94
86.62
55.85
55.62
55.52
40.01
39.85
39.68
39.51
39.34
39.18
39.01
N=CHPh
MeO
CN
8 Ar
Ar = 2,4-MeO2C6H3
200
180
160
140
120
100
80
60
40
20
0
ppm

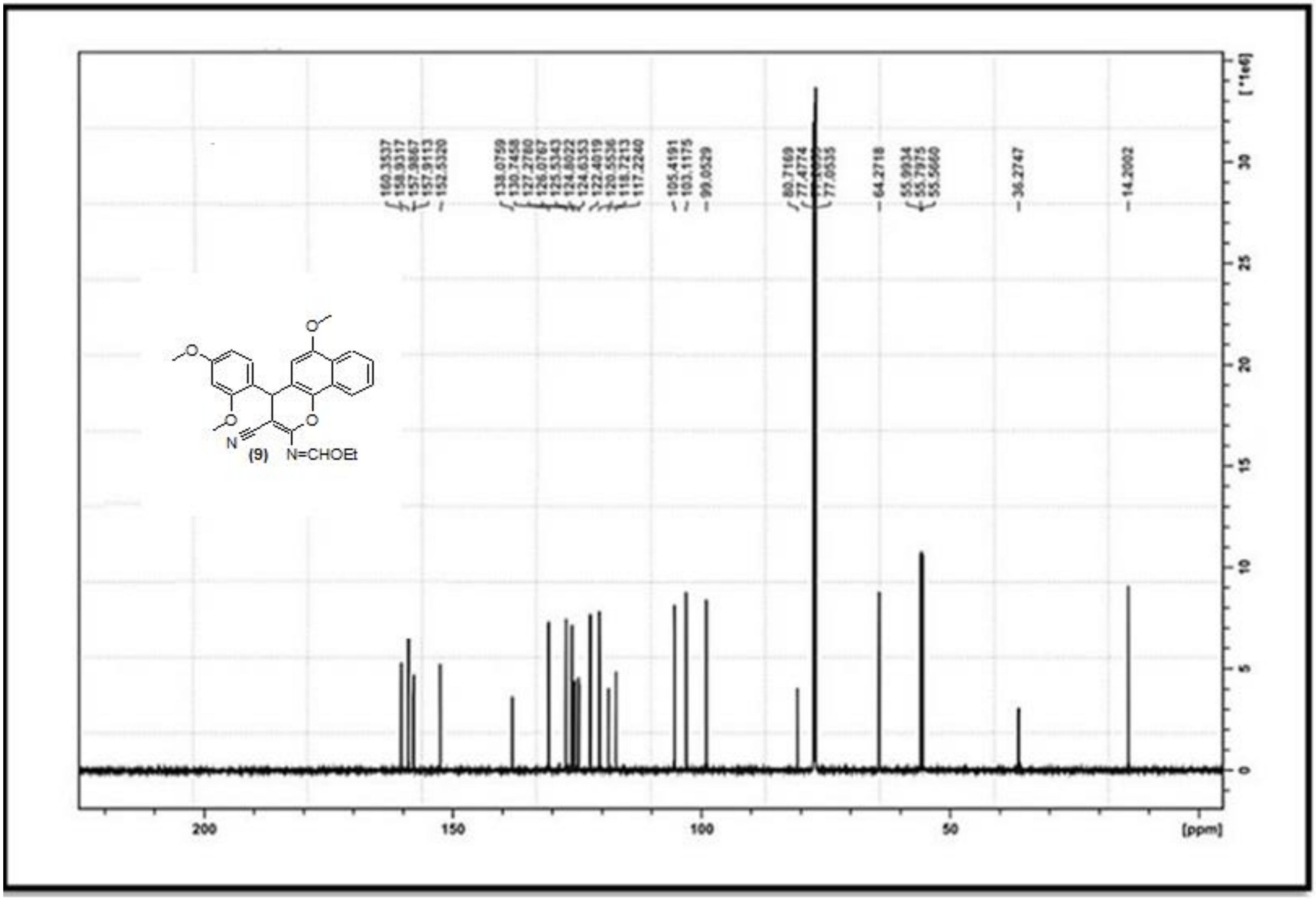
160.3537
158.9317
157.9867
157.9113
152.5320
138.0759
130.7458
127.2780
126.0767
125.5343
124.8022
124.6353
122.4019
120.5536
118.7213
117.2240
105.4191
103.1175
99.0529
80.7169
77.4774
77.0535
64.2718
55.9934
55.7975
55.5660
36.2747
14.2002
N=CHOEt
(9)
N
O
200
150
100
50
[ppm]
0
5
10
15
20
25
30
[*1e6]

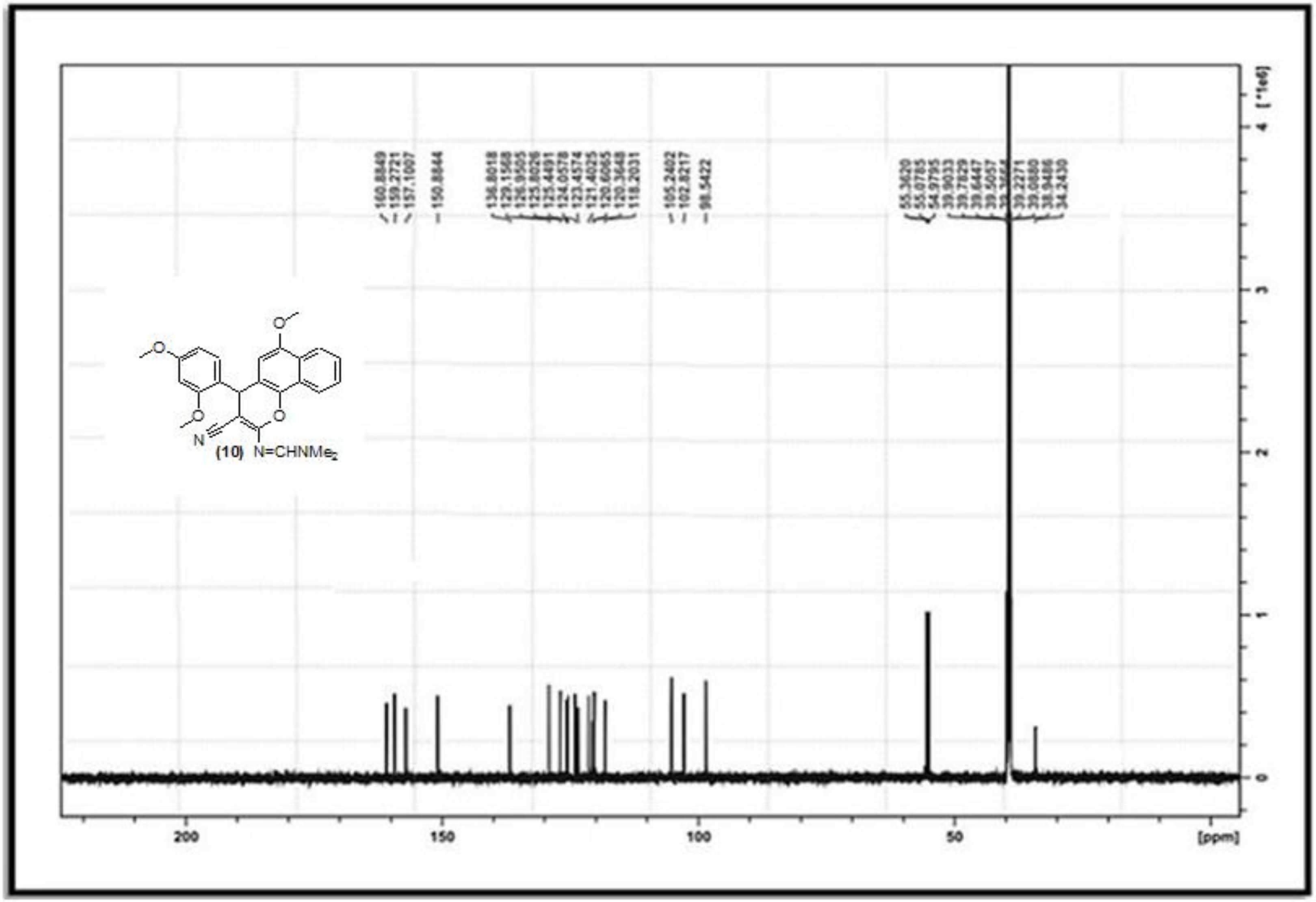

160.8849
159.2721
157.1007
150.8844
136.8018
129.1568
126.9505
125.8026
125.4491
124.0578
123.4574
121.4025
120.6065
120.3648
118.2031
105.2402
102.8217
98.5422
55.3620
55.0785
54.9795
39.9033
39.7829
39.6447
39.5057
39.2271
39.0880
38.9486
34.2430
(10) N=CHNMe2
200
150
100
50
[ppm]
[*1e6]

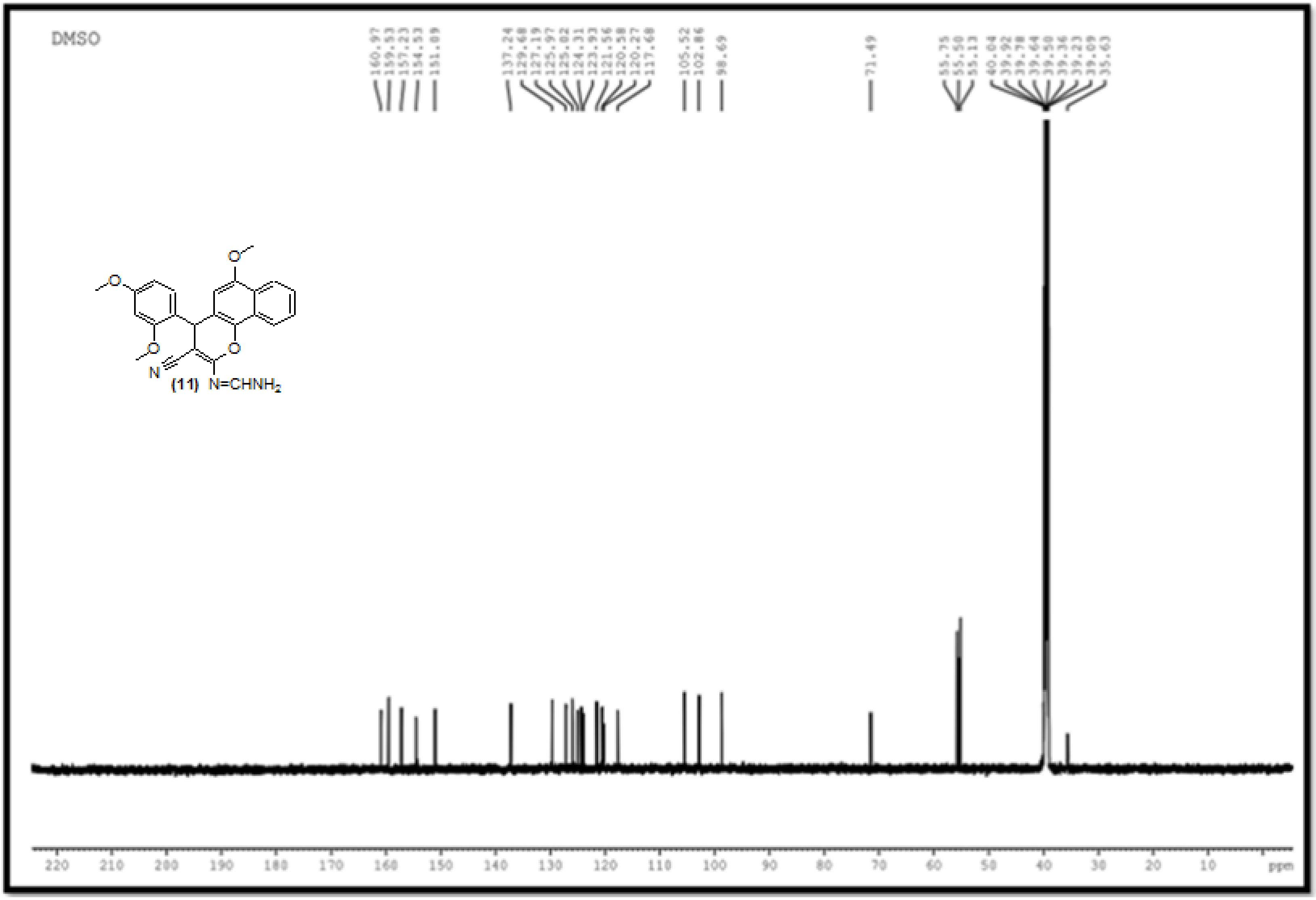
DMSO
160.97
159.53
157.23
154.53
151.09
137.24
129.68
127.19
125.97
125.02
124.31
123.93
121.56
120.58
120.27
117.68
105.52
102.86
98.69
71.49
55.75
55.50
55.13
40.04
39.92
39.78
39.64
39.50
39.36
39.23
39.09
35.63
(11) N=CHNH2
220
210
200
190
180
170
160
150
140
130
120
110
100
90
80
70
60
50
40
30
20
10
ppm

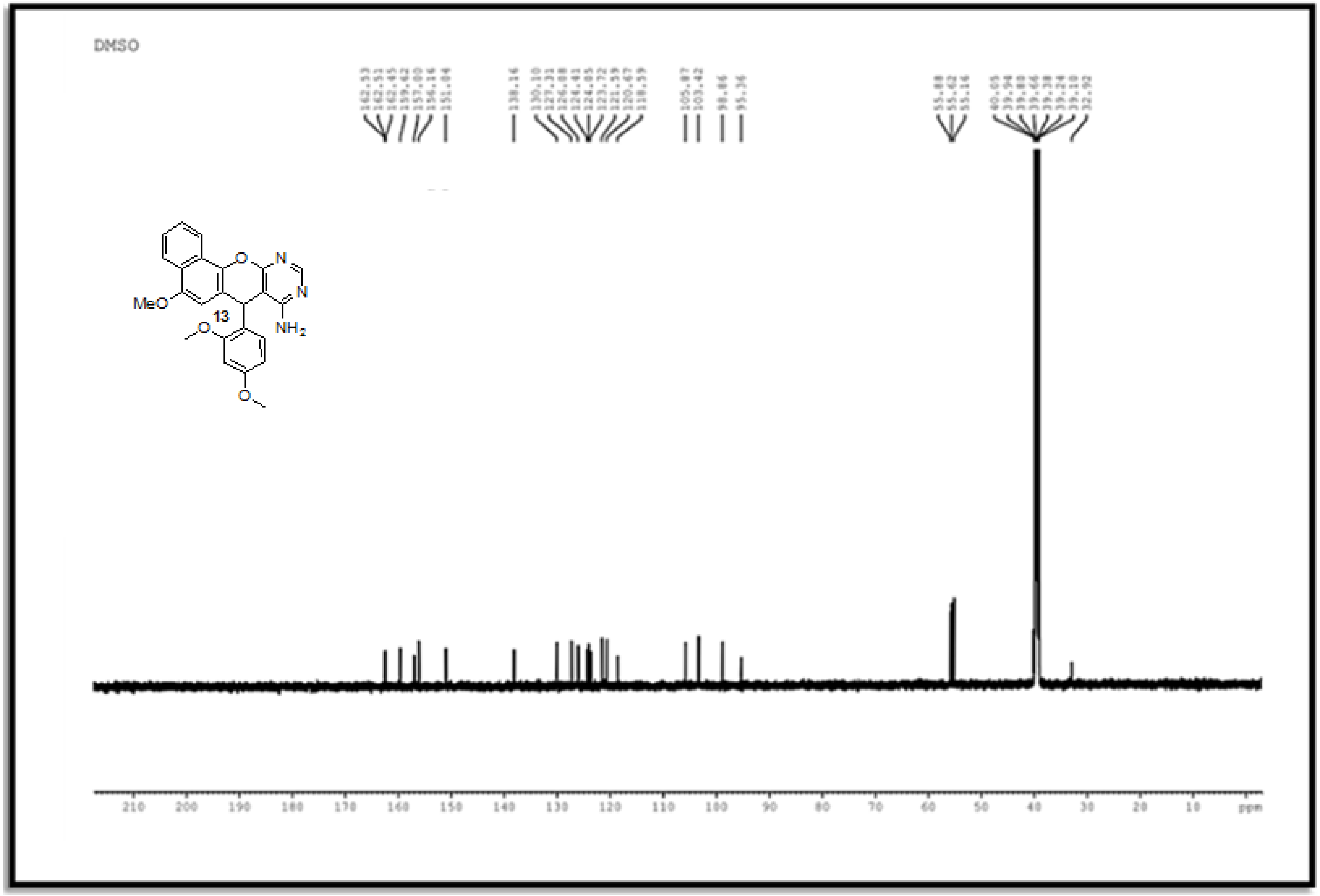
DMSO
162.53
162.51
162.45
159.42
157.00
156.16
151.04
138.16
130.10
127.31
126.08
124.41
124.05
123.72
121.59
120.67
118.59
105.87
103.42
98.86
95.36
55.88
55.62
55.16
40.05
39.94
39.80
39.66
39.38
39.24
39.10
32.92
MeO
13
NH2
ppm

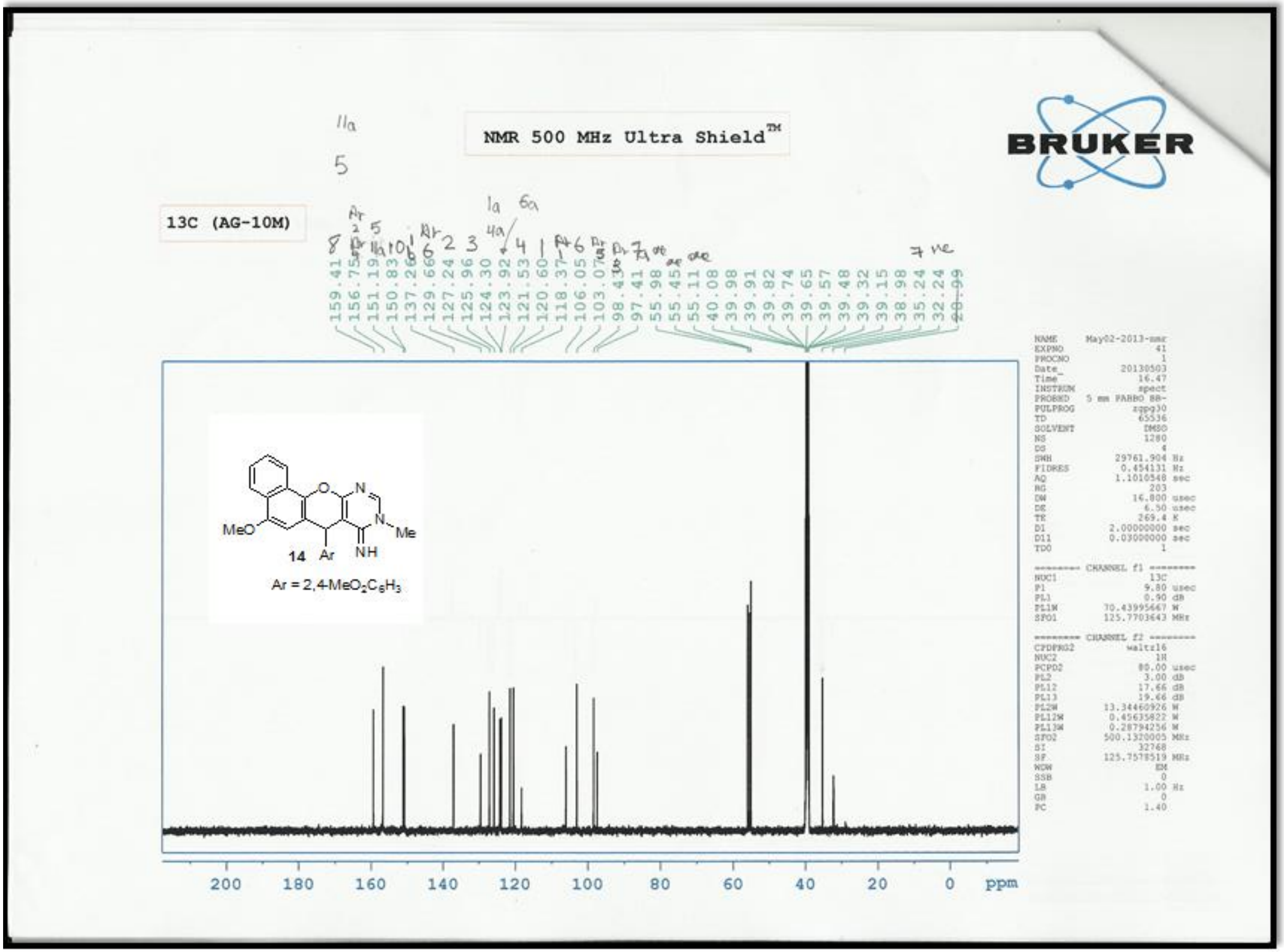
NMR 500 MHz Ultra Shield™
BRUKER
13C (AG-10M)
159.41
156.75
151.19
150.83
137.26
129.66
127.24
125.96
124.30
123.92
121.53
120.60
118.37
106.05
103.07
98.43
97.41
55.98
55.45
55.11
40.08
39.98
39.91
39.82
39.74
39.65
39.57
39.48
39.32
39.15
38.98
35.24
32.24
MeO
N
O
N
Me
NH
14
Ar
Ar = 2,4-MeO$_2$C$_6$H$_3$
NAME May02-2013-nmr
EXPNO 41
PROCNO 1
Date_ 20130503
Time 16.47
INSTRUM spect
PROBHD 5 mm PABBO BB-
PULPROG zgpg30
TD 65536
SOLVENT DMSO
NS 1280
DS 4
SWH 29761.904 Hz
FIDRES 0.454131 Hz
AQ 1.1010548 sec
RG 203
DW 16.800 usec
DE 6.50 usec
TE 269.4 K
D1 2.00000000 sec
D11 0.03000000 sec
TD0 1
======== CHANNEL f1 ========
NUC1 13C
P1 9.80 usec
PL1 0.90 dB
PL1W 70.43995667 W
SFO1 125.7703643 MHz
======== CHANNEL f2 ========
CPDPRG2 waltz16
NUC2 1H
PCPD2 80.00 usec
PL2 3.00 dB
PL12 17.66 dB
PL13 19.66 dB
PL2W 13.34460926 W
PL12W 0.45635822 W
PL13W 0.28794256 W
SFO2 500.1320005 MHz
SI 32768
SF 125.7578519 MHz
WDW EM
SSB 0
LB 1.00 Hz
GB 0
PC 1.40
200
180
160
140
120
100
80
60
40
20
0
ppm

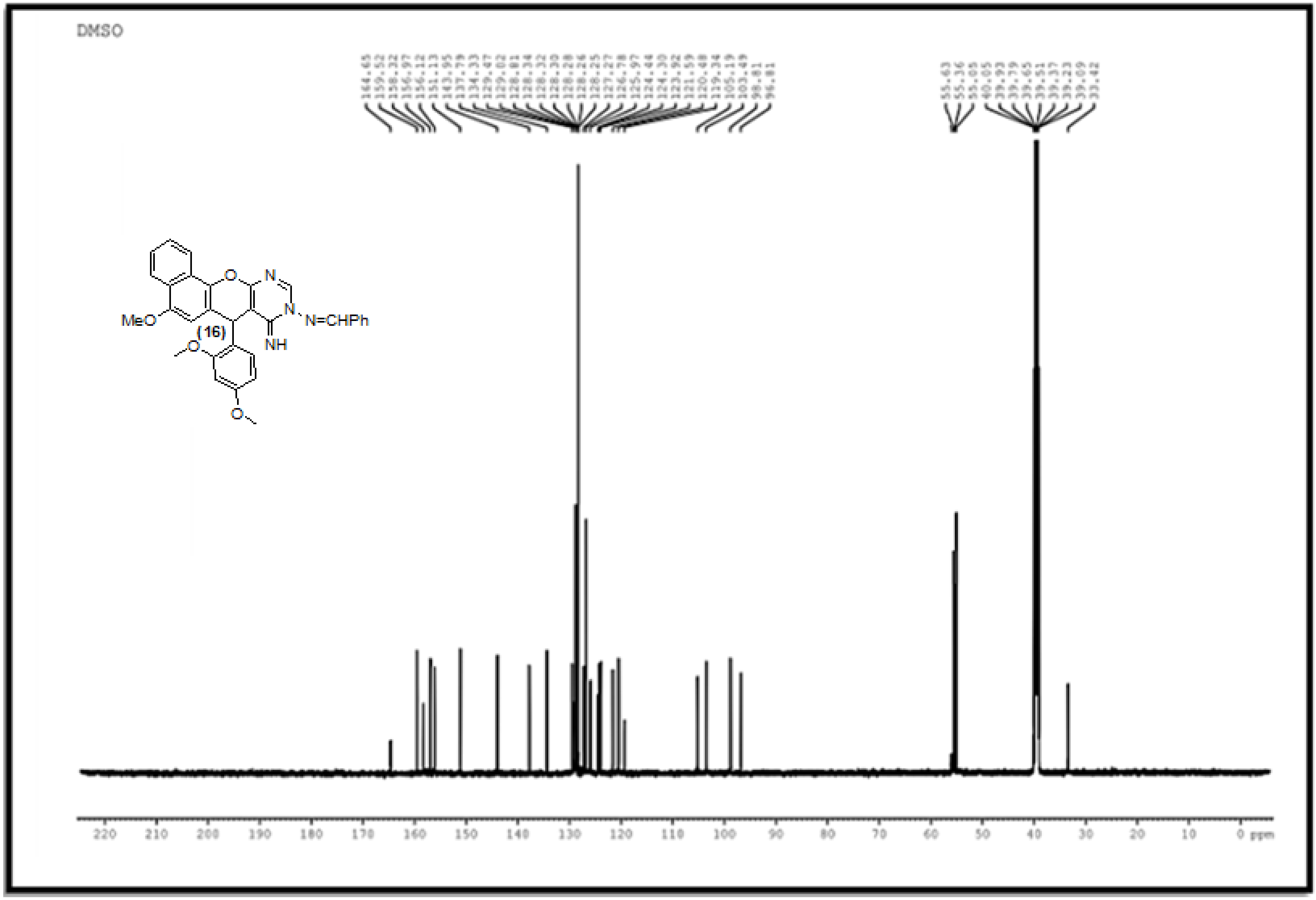
DMSO
164.65
159.52
158.32
156.97
156.12
151.13
143.95
137.79
134.33
129.47
129.02
128.81
128.34
128.32
128.30
128.28
128.26
128.25
127.27
126.78
125.97
124.44
124.30
123.92
121.59
120.48
119.34
105.19
103.49
98.81
96.81
55.63
55.36
55.05
40.05
39.93
39.79
39.65
39.51
39.37
39.23
39.09
33.42
MeO
N
O
N=CHPh
NH
(16)
220 210 200 190 180 170 160 150 140 130 120 110 100 90 80 70 60 50 40 30 20 10 0 ppm

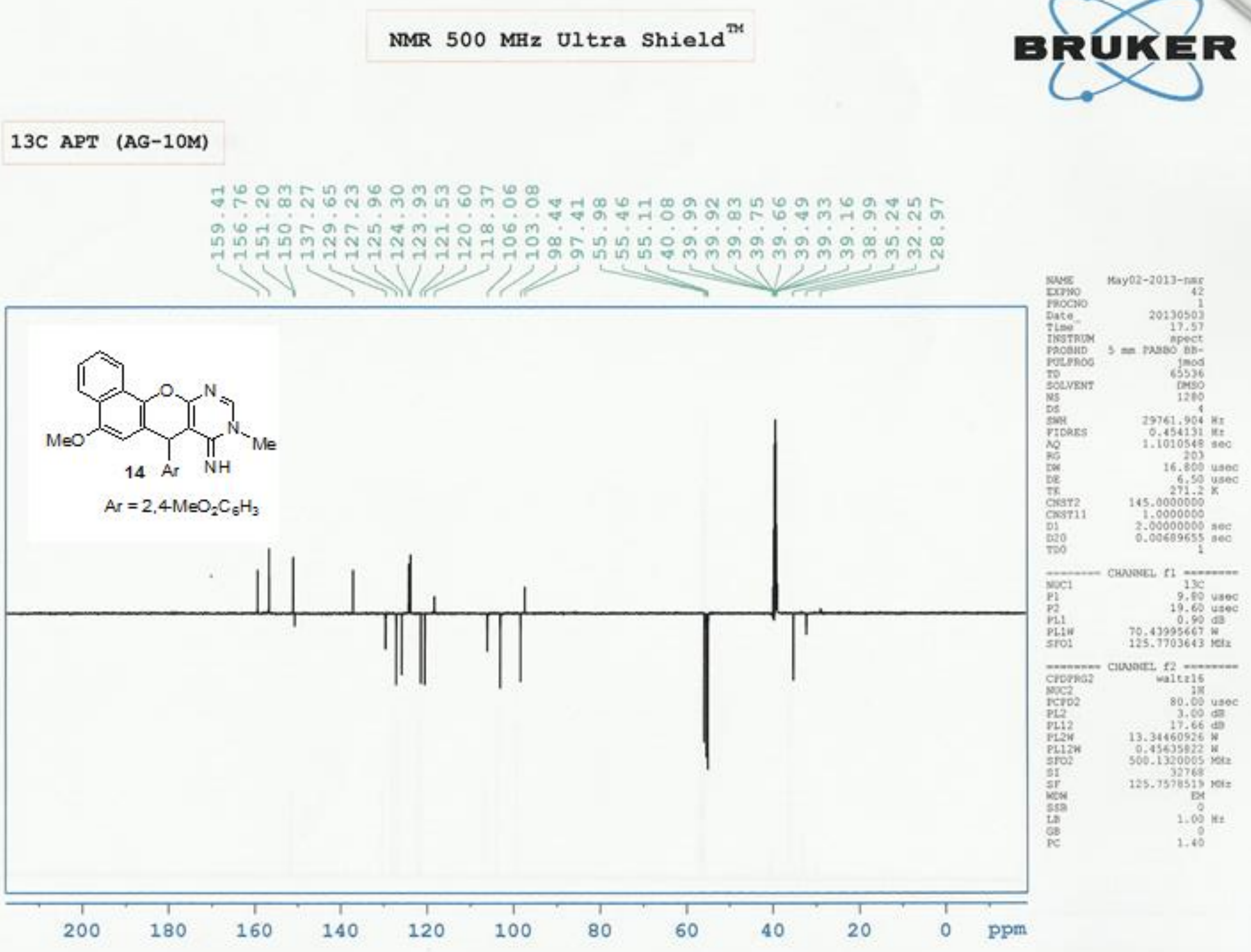
NMR 500 MHz Ultra Shield™
13C APT (AG-10M)
159.41
156.76
151.20
150.83
137.27
129.65
127.23
125.96
124.30
123.93
121.53
120.60
118.37
106.06
103.08
98.44
97.41
55.98
55.46
55.11
40.08
39.99
39.92
39.83
39.75
39.66
39.49
39.33
39.16
38.99
35.24
32.25
28.97
MeO
N
O
N
Me
14
Ar
NH
Ar = 2,4-MeO$_2$C$_6$H$_3$
200
180
160
140
120
100
80
60
40
20
0
ppm

NMR 500 MHz Ultra Shield™

13C DEPT45 (AG-10M)

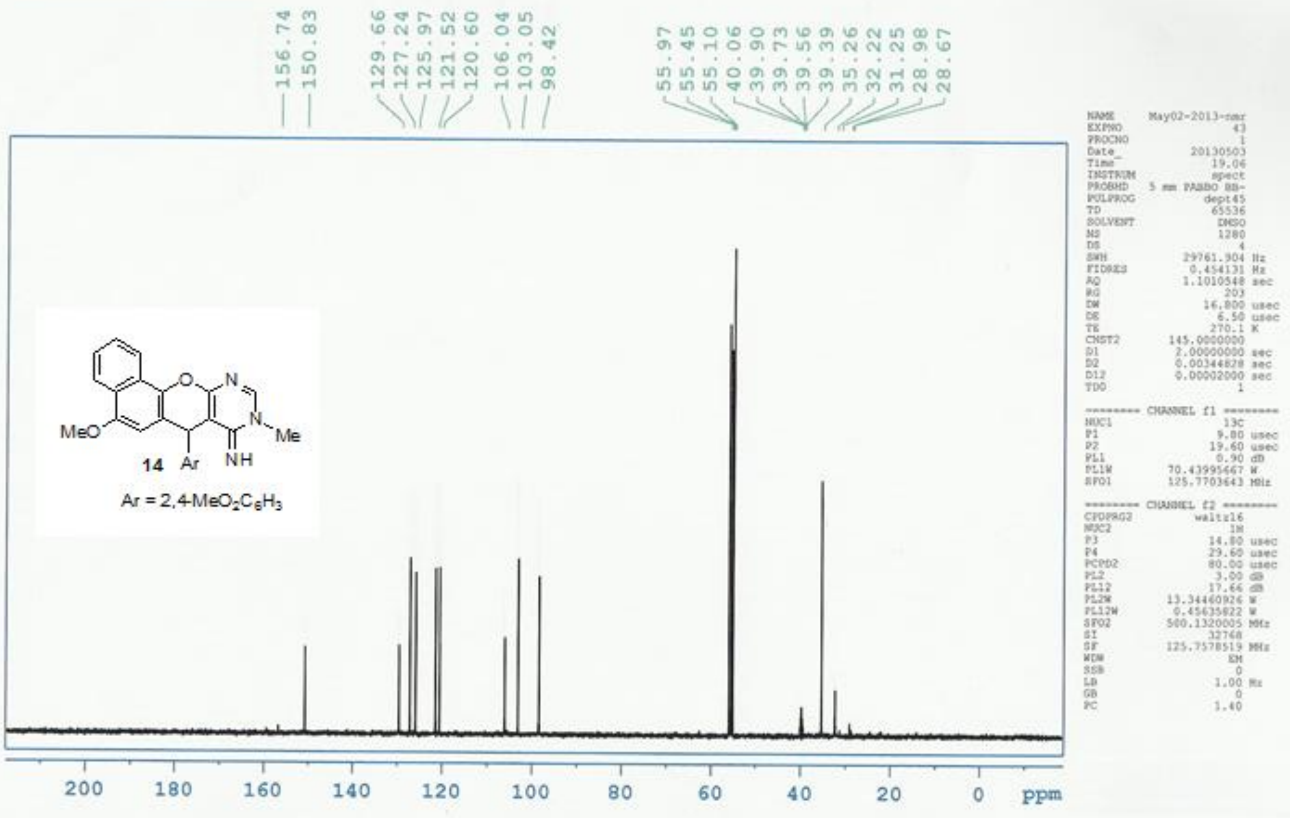

NMR 500 MHz Ultra Shield™

13C DEPT90 (AG-10M)

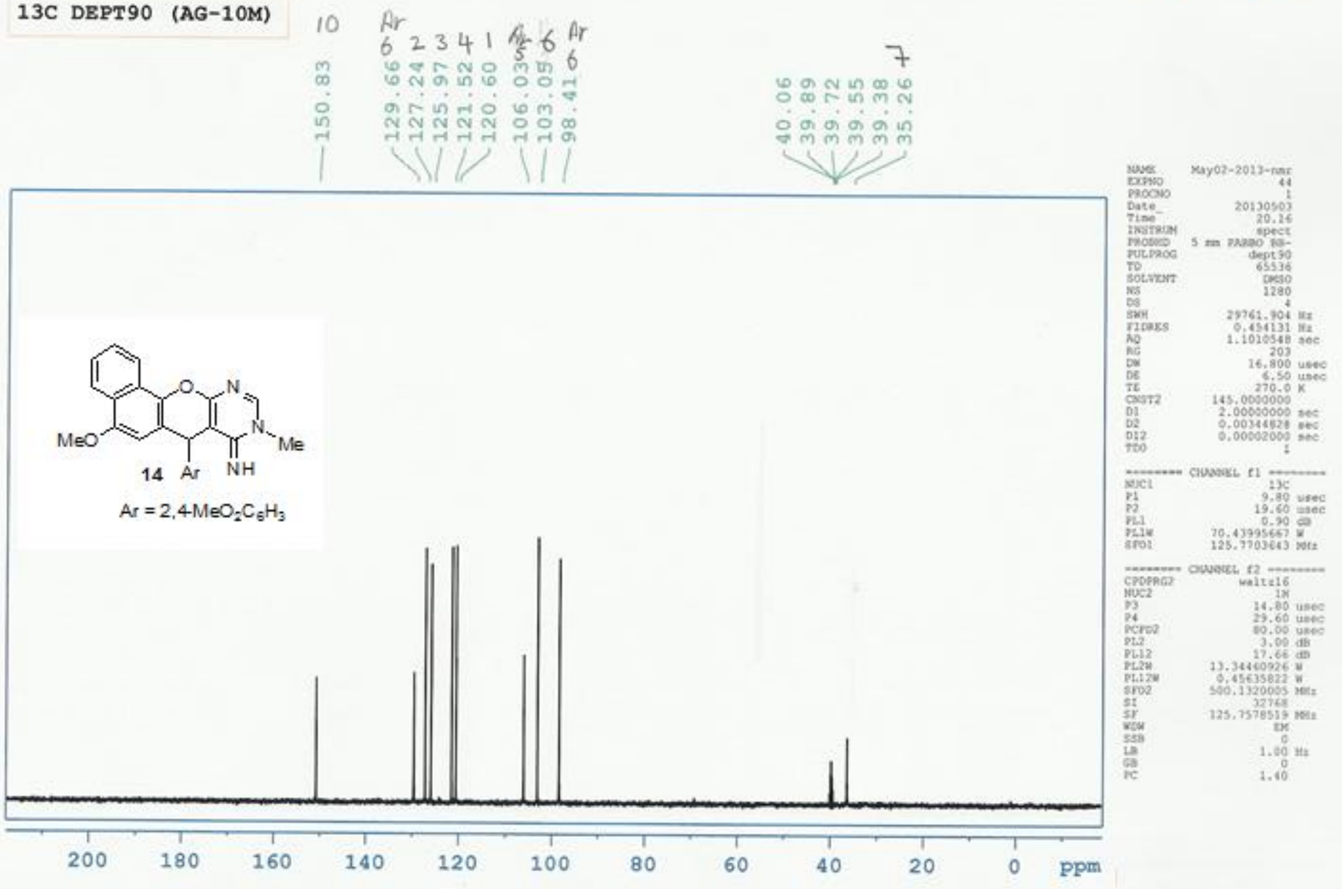

NMR 500 MHz Ultra Shield™

13C DEPT135 (AG-10M)

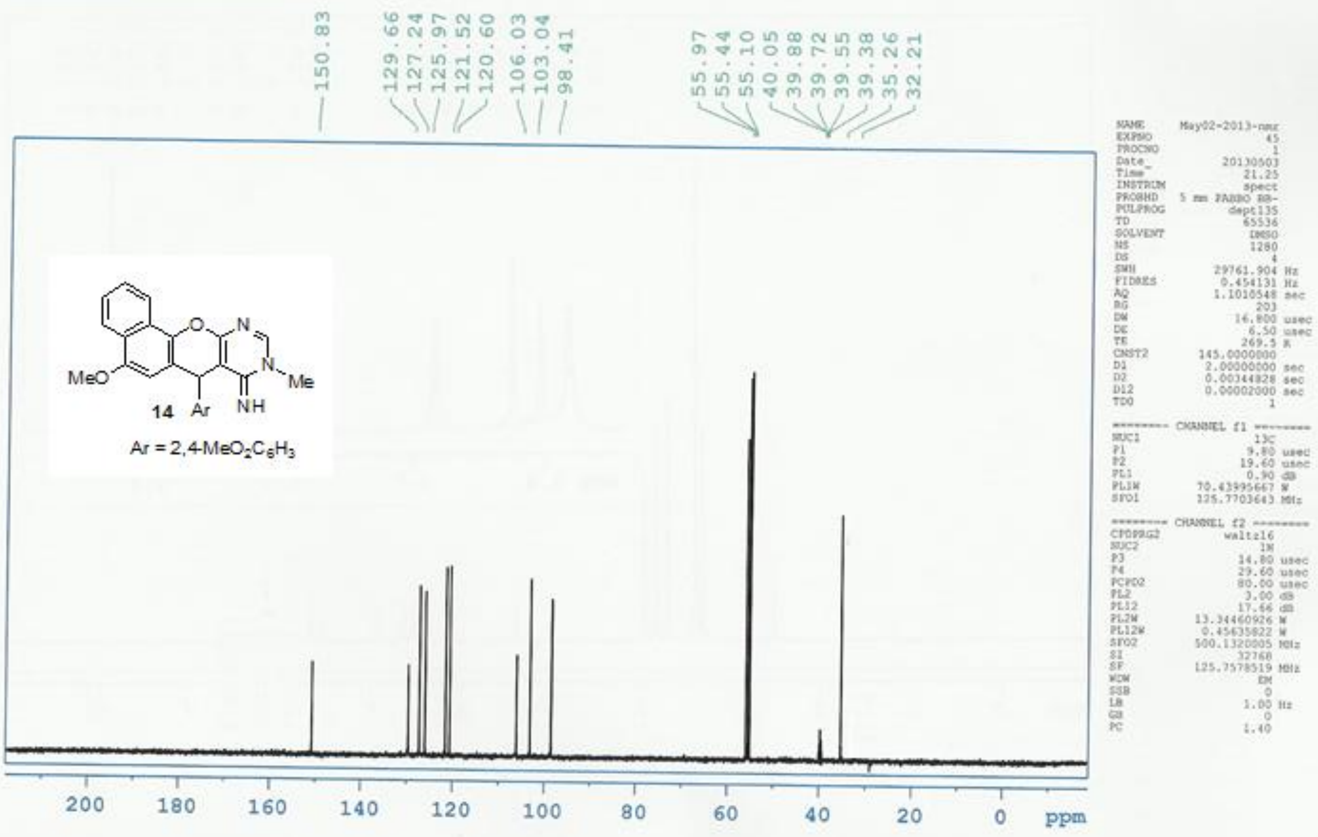

Supplement: Supplementary file 1 [file molecules-24-01060-s001.pdf]
